# Integrative multi-omics and predictive precision systems for poultry meat and egg quality: Mechanisms, applications, and commercial challenges

**DOI:** 10.1016/j.psj.2026.107378

**Published:** 2026-07-02

**Authors:** Getahun Belay Mekonnen

**Affiliations:** Department of Animal Science, Burie Campus, Debre Markos University, P.O. Box: 18, Burie, Ethiopia

**Keywords:** Adaptive systems theory of poultry quality, Artificial intelligence, Egg quality, Meat quality, Multi-omics integration

## Abstract

Poultry meat and egg quality result from complex interactions among host genetics, metabolism, nutrition, microbiome ecology, physiology, management practices, and environmental conditions. These multidimensional interactions limit the predictive capacity of conventional phenotype-based approaches and increasingly necessitate systems-level frameworks capable of capturing biological complexity. Recent advances in multi-omics technologies have transformed poultry quality research by enabling integrated analyses of genomic, transcriptomic, proteomic, metabolomic, lipidomic, epigenomic, and microbiome datasets. These approaches have substantially enhanced understanding of the molecular, cellular, physiological, and ecological networks associated with product quality, production efficiency, physiological resilience, and environmental adaptation. Integrated multi-omics analyses, particularly when combined with artificial intelligence and machine-learning approaches, have the potential to identify biologically interpretable biomarkers, candidate mechanistic pathways, and predictive signatures associated with meat and egg quality traits; however, most proposed signatures remain at early stages of validation and require rigorous external testing before commercial deployment. This review synthesizes current advances in omics-driven poultry research and critically evaluates emerging applications in precision nutrition, breeding, health monitoring, environmental adaptation, and sustainable production systems. To provide a unifying biological framework, we propose the Adaptive Systems Theory of Poultry Quality (ASTPQ), which conceptualizes poultry quality as an emergent adaptive phenotype arising from coordinated interactions among mitochondrial function, redox homeostasis, immune competence, metabolic flexibility, physiological resilience, endocrine–immune regulation, and host–microbiome dynamics. Within this conceptual framework, adaptive-system capacity is proposed as the principal integrative mechanism linking molecular regulation with phenotypic quality outcomes across diverse production environments. Despite substantial advances, commercial implementation remains constrained by biological heterogeneity, methodological variability, limited external validation, computational complexity, challenges in data integration, infrastructure requirements, and economic barriers. Current evidence suggests that predictive performance depends less on increasing molecular dimensionality than on developing biologically interpretable, externally validated, economically feasible, and operationally scalable systems. Future progress will likely require integrated precision-production frameworks that combine molecular biomarkers, physiological monitoring, environmental sensing, microbiome-informed interventions, explainable artificial intelligence, and rigorous large-scale field validation to support sustainable, resilient, and commercially applicable poultry production systems.

## Introduction

Poultry meat and eggs are among the most important animal-derived protein sources worldwide because of their production efficiency, affordability, scalability, nutritional value, and relatively low environmental footprint compared with many other livestock systems. Consequently, poultry production plays a central role in global food security, nutritional sustainability, and the provision of high-quality protein for a growing human population (FAO, 2024; Poore and Nemecek, 2018).

Advances in genetic improvement, nutritional management, disease control, housing technologies, and precision-production systems have substantially enhanced growth performance, feed efficiency, reproductive output, survivability, and overall productivity in modern poultry production (Cao et al., 2024; Havenstein et al., 2003; Zuidhof et al., 2014). Despite these advances, maintaining consistent meat and egg quality remains a major biological and commercial challenge because product quality emerges from complex interactions among genetic, physiological, microbial, nutritional, environmental, and management-related factors operating across multiple levels of biological organization.

Intensive selection for rapid growth and increased breast muscle yield in broiler chickens has been accompanied by a higher prevalence of myopathies, including wooden breast, white striping, and spaghetti meat. These conditions are characterized by disturbances in energy metabolism, mitochondrial function, vascularization, redox homeostasis, inflammatory signaling, and tissue remodeling (Abasht et al., 2016; Baldi et al., 2021; Emambu et al., 2023; Greene et al., 2025). In laying hens, reproductive aging, thermal stress, nutritional imbalances, and storage conditions negatively impact eggshell integrity, albumen function, yolk stability, and overall quality by altering calcium metabolism, biomineralization, oxidative status, cellular physiology, and post-lay physicochemical processes (Chang et al., 2024a, b; Feng et al., 2023; Fu et al., 2024; Gao et al., 2025b). Taken together, these observations suggest that the quality of poultry products is not determined by individual biological pathways, but rather by the combined behavior of interconnected adaptive systems.

Growing evidence from genomics, epigenomics, transcriptomics, proteomics, metabolomics, lipidomics, microbiomics, and other systems biology disciplines suggests that dynamic interactions among molecular, physiological, microbial, and environmental networks regulate poultry quality, rather than the actions of individual genes or pathways (Berri et al., 2019; Ferrocino et al., 2023; Gao et al., 2025b; Ghafouri et al., 2021; Kang et al., 2022). These interactions influence muscle development, eggshell formation, nutrient partitioning, oxidative stability, immune competence, stress resilience, and postmortem product characteristics (Fu et al., 2024; Gao et al., 2024; Gu et al., 2024; He et al., 2023). Consequently, poultry quality is increasingly recognized as an emergent, systems-level phenotype that arises from the coordinated regulation of molecular, cellular, physiological, microbial, and environmental domains.

Recent advances in high-throughput omics technologies have transformed our ability to characterize these regulatory networks at unprecedented resolution. These technologies include genomics, transcriptomics, epigenomics, proteomics, metabolomics, lipidomics, metagenomics, and single-cell multi-omics approaches, which now enable comprehensive characterization of the molecular and cellular architecture underlying poultry production and product quality (Cao et al., 2025; Conesa and Beck, 2019; Ferrocino et al., 2023; Fu et al., 2022; Goossens et al., 2022; Hasin et al., 2017). Integration of these complementary data layers facilitates biomarker discovery, strengthens mechanistic understanding, and supports the development of predictive frameworks linking biological signatures to economically important quality traits. Concurrent advances in computational biology, AI, ML, network science, and systems modeling further enhance the extraction of biologically meaningful information from high-dimensional datasets, accelerating the development of predictive and translational applications for precision poultry production (Chen et al., 2025b; Dhungana et al., 2025; Essien et al., 2026).

Despite these advances, existing reviews have generally focused on individual omics disciplines, specific quality traits, or isolated aspects of poultry production. To our knowledge, no previous review has comprehensively integrated poultry meat quality, egg quality, multi-omics technologies, predictive biology, commercial translation, and adaptive systems theory within a unified conceptual framework. While systems-biology principles have previously been applied in animal science, the proposed Adaptive Systems Theory of Poultry Quality (ASTPQ) represents a novel conceptual synthesis that integrates current evidence to explain poultry product quality as an emergent property of adaptive-system function across multiple biological levels. Accordingly, this review has three primary objectives: (i) to critically synthesize current evidence on the biological regulation of poultry meat and egg quality from an integrative multi-omics perspective; (ii) to evaluate the biological interpretability, translational readiness, reproducibility, and commercial applicability of emerging predictive approaches; and (iii) to present the ASTPQ framework as a unifying conceptual model that generates testable hypotheses to guide future mechanistic, predictive, and translational research. The specific hypotheses derived from ASTPQ are developed in Section 5.3 and provide the conceptual framework through which the evidence synthesized throughout this review is critically interpreted.

## REVIEW FRAMEWORK AND INTEGRATIVE SCOPE

### Review Scope

This narrative review synthesizes current evidence regarding the biological mechanisms, multi-omics integration frameworks, predictive applications, and translational implications underlying poultry meat and egg quality traits. It places particular emphasis on mechanistic interpretation, critical evaluation of evidence, and the identification of opportunities and limitations associated with precision production systems. The review integrates findings across multiple biological scales to provide a comprehensive assessment of the factors governing product quality and their potential applications in commercial environments.

### Integrative Multi-Omics Framework

**Fig. 1:**
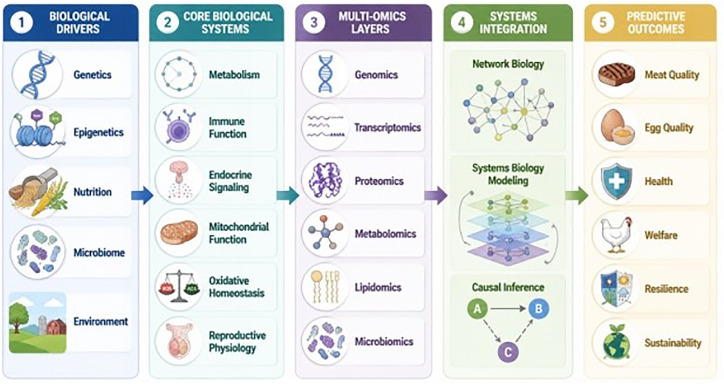
**Systems biology framework for predictive poultry meat and egg quality:** Genetic, epigenetic, nutritional, microbial, physiological, immunological, and environmental factors interact through interconnected biological networks that regulate metabolism, mitochondrial function, oxidative homeostasis, immune competence, and physiological adaptation. These processes generate complementary genomic, transcriptomic, proteomic, metabolomic, lipidomic, microbiome, phenotypic, and environmental datasets that can be integrated using systems-biology, network-analysis, and causal-inference frameworks. Integrated analyses facilitate the identification of molecular pathways, biologically interpretable biomarkers, and predictive signatures associated with meat quality, egg quality, health, welfare, resilience, and production performance.

Poultry meat and egg quality are complex phenotypes that arise from interactions among genomic, transcriptomic, proteomic, metabolomic, lipidomic, microbiome, physiological, nutritional, and environmental determinants. Understanding such variation requires integrative frameworks that link molecular processes with phenotypic outcomes across multiple levels of biological organization. Multi-omics integration provides a systems-level approach for identifying regulatory mechanisms, biologically meaningful biomarkers, and predictive relationships underlying product quality, adaptive capacity, physiological resilience, and production performance. By combining complementary layers of biological information, these approaches facilitate a more comprehensive understanding of the interconnected networks that govern poultry quality and productivity (Fig. 1; Table 1).

**Table 1:** Biological information layers and predictive contributions of multi-omics approaches in poultry meat and egg quality research.

Omics Layer	Primary Biological Information	Poultry Quality Applications	Principal Limitation	References
Genomics	Inherited genetic variation underlying complex production, quality, and adaptive traits	Genomic selection for meat yield, myopathy susceptibility, eggshell quality, and egg-production traits	Polygenic architecture and G × E interactions	Aggrey et al., 2010; Bailey et al., 2020; Chu et al., 2019
Transcriptomics	Dynamic gene-expression patterns reflecting physiological state, developmental processes, and environmental responses	Stress responses, muscle development, reproductive biology, and albumen formation	Temporal variability and tissue specificity	Beauclercq et al., 2017; Coble et al., 2014; Gu et al., 2024
Proteomics	Functional protein abundance, modification, and pathway activity	Muscle structure, postmortem meat quality, and eggshell-matrix biology	High analytical cost and technical complexity	Dai et al., 2025; Gautron et al., 2021
Metabolomics	Metabolic-state profiling reflecting physiological function and biochemical activity	Flavor development, oxidative stability, nutrient utilization, and stress adaptation	Environmental sensitivity and context-dependent interpretation	Beauclercq et al., 2018; Ge et al., 2022; Greene et al., 2020
Lipidomics	Lipid composition, signaling, and oxidative metabolism	Fatty-acid composition, lipid oxidation, omega-3 enrichment, and yolk quality	Limited standardization and methodological variability	Chin et al., 2023; Guo et al., 2024b
Microbiome Profiling	Host–microbiome interactions influencing metabolism, immunity, and physiological regulation	Gut health, nutrient efficiency, immune resilience, and environmental adaptation	Temporal instability and limited causal inference	Bernard et al., 2024; Dai et al., 2022; Gao et al., 2017
Integrated Multi-Omics	Cross-layer integration of genomic, transcriptomic, proteomic, metabolomic, microbial, phenotypic, and environmental information	Systems-level prediction of meat quality, egg quality, resilience, and production stability	Data integration complexity, high dimensionality, and external-validation requirements	An et al., 2025; Cui et al., 2024

Footnote: Omics layers provide complementary biological information for elucidating and predicting poultry meat and egg quality traits. Predictive utility varies according to genetic architecture, environmental conditions, and analytical methodology. G × E, genotype-by-environment interaction.

### Evidence Evaluation and Translational Perspective

This narrative review synthesizes current evidence on the biological mechanisms, multi-omics integration strategies, predictive applications, and translational implications of poultry meat and egg quality. Evidence was critically evaluated according to biological plausibility, methodological rigor, reproducibility, validation stage, translational relevance, and practical applicability. Particular emphasis was placed on distinguishing experimentally supported causal mechanisms from predictive associations and on differentiating externally validated findings from proof-of-concept technologies. Because the available literature exhibits substantial heterogeneity in experimental design, poultry populations, phenotyping methods, analytical platforms, bioinformatics workflows, and reporting standards, quantitative meta-analysis was not considered appropriate. Accordingly, the conclusions presented in this review preferentially emphasize findings replicated across multiple independent investigations while explicitly acknowledging areas where evidence remains preliminary, context-dependent, or predominantly correlative.

## Integrated multi-omics of meat quality

### Molecular regulation of meat quality

Poultry meat quality is a complex, multifactorial phenotype shaped by interactions among metabolic, mitochondrial, calcium-regulatory, redox, vascular, immune, extracellular-matrix, and postmortem biochemical processes (Bottje, 2019; Chen et al., 2022; Ding et al., 2026; Greene et al., 2025). Integrated transcriptomic, proteomic, metabolomic, lipidomic, and epigenomic investigations demonstrate that economically important traits, including tenderness, water-holding capacity, color stability, oxidative resistance, pH decline, processing yield, flavor development, and storage performance, are governed by coordinated regulation across interconnected molecular and physiological networks rather than by isolated biological mechanisms (Cui et al., 2024; Ding et al., 2026; Ghafouri et al., 2021; Hao et al., 2025). Accordingly, meat quality is increasingly recognized as an adaptive systems-level phenotype that emerges from dynamic interactions among genetic variation, metabolic regulation, environmental influences, and postmortem biochemical processes.

Genetic selection for rapid growth, enhanced feed efficiency, and increased breast-muscle yield has substantially improved poultry productivity. However, it has also contributed to a higher prevalence of growth-related myopathies, including wooden breast, white striping, and spaghetti meat (Abasht et al., 2016; Emambu et al., 2023; Greene et al., 2025). Multi-omics investigations consistently demonstrate that these conditions are associated with disruptions in mitochondrial bioenergetics, hypoxia responses, oxidative balance, calcium homeostasis, inflammatory signaling, vascular function, and extracellular-matrix remodeling (Chen et al., 2022; Ding et al., 2026; Greene et al., 2020; 2025). These alterations are accompanied by impaired oxidative phosphorylation, metabolic dysregulation, fibrosis, and activation of cellular stress pathways that collectively compromise muscle integrity, processing performance, sensory characteristics, and overall product quality (Ding et al., 2026; Greene et al., 2025). Such findings highlight the physiological trade-offs that may arise when genetic improvement exceeds the adaptive capacity of underlying biological systems.

The transformation of living muscle into meat is governed by coordinated postmortem processes. These involve glycolysis, adenosine triphosphate depletion, calcium mobilization, proteolysis, mitochondrial metabolism, and oxidative reactions that collectively determine pH decline, protein functionality, water retention, tenderness, texture, color stability, flavor development, and shelf life (Bottje, 2019; Chen et al., 2022; Ge et al., 2022). Metabolomic analyses have identified pathways associated with amino-acid metabolism, energy production, redox homeostasis, and flavor-precursor generation. Lipidomic investigations highlight the importance of phospholipid composition, fatty-acid metabolism, membrane integrity, and susceptibility to lipid oxidation in determining sensory quality, oxidative stability, nutritional value, and storage performance (Cui et al., 2024; Ge et al., 2022; Hao et al., 2025). Taken together, these findings highlight the central role of metabolic regulation in shaping meat-quality outcomes throughout production, processing, and postmortem storage.

Accumulating evidence indicates that gut microbial communities contribute to variations in meat quality by influencing nutrient utilization, intestinal barrier integrity, immune regulation, microbial metabolite production, and systemic metabolic homeostasis (Bernard et al., 2024; Calik et al., 2022; He et al., 2023). Microbial metabolites, particularly short-chain fatty acids (**SCFAs**) and other bioactive compounds, affect energy metabolism, inflammatory regulation, nutrient partitioning, and tissue development, thereby linking gut ecology with muscle growth, carcass composition, and product quality traits (Calik et al., 2022; Gautam et al., 2024; He et al., 2023). However, most microbiome-associated relationships remain predominantly correlative. Therefore, robust causal inference will require controlled intervention studies, longitudinal experimental designs, mechanistic validation, and cross-population replication to establish reproducible biomarkers and facilitate commercial application (Brugaletta et al., 2023a, Brugaletta et al., 2023b; Gautam et al., 2024; Haas et al., 2023).

### Predictive multi-omics models

Predictive multi-omics approaches integrate genomic, epigenomic, transcriptomic, proteomic, metabolomic, lipidomic, microbiome, physiological, and environmental data to identify molecular signatures associated with poultry meat-quality traits before overt phenotypic deterioration becomes apparent. This integration enables earlier risk assessment, targeted intervention, and more effective precision-management strategies (Cui et al., 2024; Ding et al., 2026; Greene et al., 2025; Hao et al., 2025). Unlike conventional phenotype-based assessments, which typically detect quality defects only after deterioration has occurred, multi-omics frameworks capture molecular and physiological alterations throughout trait development. This provides mechanistic insight into the pathways underlying growth-related myopathies, oxidative instability, impaired processing performance, and responses to nutritional and environmental stressors (Cui et al., 2024; Ding et al., 2026; Greene et al., 2025). Collectively, these advances support a transition from reactive quality control toward proactive, mechanism-based quality management.

Advances in ML, AI, network biology, and computational systems modeling have substantially enhanced the integration of high-dimensional biological datasets and the extraction of biologically meaningful information from complex molecular interactions (Chen et al., 2025b; Cui et al., 2024; Doornweerd et al., 2024; Essien et al., 2026). Supervised-learning algorithms, deep-learning architectures, network-based approaches, and multi-layer integration strategies facilitate the identification of biomarkers, regulatory pathways, interaction networks, and latent features associated with variation in meat-quality traits (Conesa and Beck, 2019; Cui et al., 2024; Ding et al., 2026; Hasin et al., 2017). Integrative models that combine transcriptomic, metabolomic, proteomic, lipidomic, and physiological information have improved the detection of wooden breast, metabolic dysfunction, oxidative deterioration, and stress-related quality defects while providing greater mechanistic insight than single-omics analyses alone (Ding et al., 2026; Greene et al., 2025; Hao et al., 2025). These findings demonstrate that integration across complementary biological layers enhances both predictive performance and mechanistic understanding.

Longitudinal profiling further strengthens model performance by capturing temporal molecular and physiological responses to developmental, nutritional, environmental, and management-related influences throughout the production cycle (Brugaletta et al., 2023a,b; Essien et al., 2026). Repeated measurements characterize biological trajectories rather than isolated observations, enabling earlier detection of disturbances associated with muscle pathology, oxidative imbalance, metabolic dysfunction, and stress responses (Essien et al., 2026; Wolthuis et al., 2023). Such approaches improve understanding of disease progression, adaptive responses, metabolic resilience, and recovery processes while expanding opportunities for intervention before irreversible quality deterioration occurs. Consequently, longitudinal analyses provide a more biologically informative framework for evaluating dynamic changes in health and product-quality outcomes than static end-point measurements.

Investigations using a network-based approach suggest that predictive performance improves when interactions among genes, proteins, metabolites, microbial communities, and physiological traits are incorporated into analytical frameworks instead of being evaluated independently (Ghafouri et al., 2021; Haas et al., 2023; He et al., 2023). This systems-oriented approach facilitates identifying regulatory hubs, causal pathways, interaction networks, and emergent biological properties that single-layer analyses may miss. Together, these findings support the idea that the quality of poultry meat arises from the coordinated action of molecular, physiological, microbial, and environmental processes operating at various levels of biological organization.

Despite substantial progress, most multi-omics prediction frameworks are still in the experimental, proof-of-concept, or early validation stages. Relatively few studies have demonstrated consistent performance across different populations, genetic backgrounds, management systems, and commercial production environments (Doornweerd et al., 2024; Essien et al., 2026). Limitations include small sample sizes, heterogeneous phenotyping protocols, incomplete data integration, model overfitting, inadequate external validation, limited mechanistic interpretability, and inconsistent reproducibility across production settings (Conesa and Beck, 2019; Goossens et al., 2022; Hasin et al., 2017). Furthermore, many models prioritize statistical accuracy while offering limited insight into the biological mechanisms underlying predictions. This constrains their practical application in breeding programs and precision management systems.

Future research should prioritize the following: large-scale, multi-population validation; standardized phenotyping protocols; harmonized data integration pipelines; longitudinal evaluation; causal inference methodologies; and interpretable analytical frameworks suitable for commercial deployment. Particular emphasis should be placed on developing cost-effective biomarker panels, integrating genomic selection with multi-omics data, and establishing scalable decision support platforms that can be routinely implemented in precision production systems and breeding programs (Chen et al., 2025b; Hidalgo et al., 2021; Hidalgo et al., 2023). The greatest value of multi-omics prediction ultimately lies in its capacity to reveal actionable biological mechanisms that support sustainable improvements in poultry meat quality, in addition to improved forecasting accuracy.

### Precision nutrition and environmental modulation

Precision nutrition represents an adaptive management strategy that integrates molecular biomarkers, metabolomic profiles, physiological indicators, microbiome-derived metabolites, genomic information, environmental measurements, and production-performance data to optimize nutrient delivery according to dynamic biological requirements rather than population-based nutritional averages (Cao et al., 2024; Essien et al., 2026; Ferrocino et al., 2023; Gao et al., 2024). This approach seeks to improve nutrient-use efficiency, metabolic adaptability, physiological resilience, growth performance, feed conversion efficiency, and meat quality while reducing losses and enhancing sustainability by accounting for genetic background, metabolic status, gut microbial ecology, physiological condition, and environmental influences (Gao et al., 2024; Ghasemi et al., 2025; Goossens et al., 2022). Consequently, nutritional requirements are increasingly recognized as dynamic, context-dependent traits shaped by interactions among host genetics, physiology, microbiome composition, environmental conditions, and production objectives.

Heat stress remains one of the most significant determinants of meat-quality variation among environmental stressors. It disrupts intestinal barrier integrity, mitochondrial function, metabolic homeostasis, redox balance, endocrine regulation, immune responses, and nutrient utilization (Akbarian et al., 2016; Chaudhary and Mishra, 2024; Chen et al., 2022; Emami et al., 2021). These disturbances contribute to reduced growth performance, impaired feed efficiency, altered carcass composition, oxidative damage, compromised processing characteristics, and deterioration of both sensory and technological quality attributes (Chen et al., 2022; Emami et al., 2021; Mancinelli et al., 2023). Multi-omics investigations further demonstrate that heat stress induces coordinated transcriptomic, proteomic, metabolomic, epigenomic, microbiome, and physiological responses involving oxidative stress, inflammation, mitochondrial dysfunction, altered lipid metabolism, calcium dysregulation, and cellular pathways that collectively impair meat quality under commercial production conditions (An et al., 2025; Chaudhary and Mishra, 2024; Coble et al., 2014). These findings highlight heat stress as a systems-level biological challenge that affects interconnected regulatory networks rather than a single environmental perturbation.

Targeted nutritional interventions can mitigate many adverse effects by modulating gut microbial ecology, intestinal integrity, immune function, mitochondrial efficiency, energy metabolism, antioxidant defenses, and redox regulation (Calik et al., 2022; Deng et al., 2022; Elbaz et al., 2023; Elokil et al., 2024). Experimental evidence supports the beneficial effects of probiotics, prebiotics, synbiotics, phytogenic compounds, functional amino acids, trace minerals, selenium, vitamin E, omega-3 fatty acids, and other antioxidant-based strategies in enhancing physiological resilience and preserving meat quality under environmental stress conditions (Calik et al., 2022; Deng et al., 2022; Dev et al., 2020; Elbaz et al., 2023; Elgendey et al., 2022; Hashemitabar and Hosseinian, 2024). Accumulating evidence further indicates that the efficacy of these interventions depends not only on nutrient composition but also on their capacity to modulate host–microbiome–metabolism interactions that govern growth, immunity, oxidative balance, metabolic flexibility, and tissue development (Brugaletta et al., 2023a,b; Ferrocino et al., 2023; He et al., 2023).

An emerging translational strategy combines genomic information, physiological biomarkers, microbiome profiles, environmental sensor outputs, automated monitoring technologies, and production records to develop genotype-informed and phenotype-informed feeding systems capable of dynamically aligning nutrient supply with biological demand throughout the production cycle (Chu et al., 2019; Doornweerd et al., 2024; Essien et al., 2026; Gao et al., 2024). Advances in sensor technologies, AI, ML, automated environmental monitoring, and real-time decision-support systems increasingly enable continuous assessment of bird health, environmental conditions, nutritional status, and production performance, creating opportunities for adaptive nutritional management under commercial conditions (Dhungana et al., 2025; Elwakeel, 2025; Essien et al., 2026). Collectively, these approaches have considerable potential to improve resource-use efficiency, environmental sustainability, animal welfare, production resilience, and the consistency of meat-quality outcomes across diverse environments.

Despite these advances, limited biomarker validation, difficulties associated with integrating heterogeneous datasets, computational and infrastructure requirements, variability among production systems, workforce-training demands, economic considerations, and insufficient large-scale field validation (Essien et al., 2026; Goossens et al., 2022) continue to constrain broad commercial adoption. Furthermore, many proposed frameworks remain largely experimental, and relatively few studies have demonstrated consistent biological, operational, and economic benefits under commercial production conditions.

Future progress will require standardized analytical workflows, harmonized phenotyping strategies, harmonized data standards, validated biomarker panels, multi-environment evaluation, interpretable predictive models, scalable digital infrastructures, and comprehensive cost–benefit assessments that evaluate both biological efficacy and economic viability. Particular emphasis should be placed on integrating causal biological understanding with predictive analytics to ensure that nutritional interventions are scientifically justified, operationally feasible, economically sustainable, and commercially relevant. Ultimately, successful implementation will depend on the development of integrated precision-production systems capable of translating biological knowledge into practical strategies that consistently enhance poultry health, productivity, sustainability, and meat quality across diverse production environments.

## Integrated multi-omics of egg quality

### Molecular regulation of egg quality

Egg quality is a complex, multifactorial phenotype shaped by interactions among reproductive, metabolic, endocrine, immune, genetic, microbial, and environmental processes. These collectively regulate eggshell formation, albumen functionality, yolk composition, oxidative stability, nutrient deposition, and embryonic support (Feng et al., 2023; Fu et al., 2024; Gao et al., 2025b; Garcia-Mejia et al., 2024; Guo et al., 2024b). Accumulating evidence indicates that egg-quality traits emerge from coordinated regulation across molecular, cellular, tissue, physiological, and environmental levels of biological organization rather than from isolated pathways. Accordingly, understanding variation in egg quality requires the integration of reproductive physiology with multi-omics, metabolic, and environmental perspectives.

The formation of eggs depends on the sequential activity of specialized oviduct compartments that regulate albumen secretion, shell membrane formation, eggshell biomineralization, pigment deposition, and cuticle development. These compartments determine the characteristics of the final product, such as its structure, nutrition, function, and protection (Feng et al., 2020; 2023; Fu et al., 2024; Gautron et al., 2021). The ovary governs follicular development, yolk precursor synthesis, and nutrient deposition. The magnum produces albumen proteins; the isthmus forms shell membranes; and the uterus (shell gland) regulates biomineralization by coordinating calcium transport, ion regulation, matrix protein secretion, and crystal growth (Cui et al., 2021; 2025; Feng et al., 2023; Fu et al., 2024). These processes are integrated through endocrine signaling, metabolic regulation, mineral homeostasis, mitochondrial function, and physiological adaptation. Together, these factors influence egg quality traits throughout the laying cycle (Chang et al., 2024a, b; Feng et al., 2023).

Reproductive aging is among the most important determinants of commercial egg quality. Longitudinal investigations demonstrate that advancing age influences shell strength, thickness, ultrastructure, albumen height, Haugh-unit values, yolk composition, oxidative stability, hatchability, and storage performance (Chang et al., 2024a,b; Feng et al., 2020; Fu et al., 2024; Gao et al., 2025b; Gu et al., 2021). These changes are associated with progressive alterations in calcium metabolism, mitochondrial function, epithelial transport capacity, endocrine regulation, antioxidant defenses, and reproductive-tissue secretory activity (Chang et al., 2024a,b; Fu et al., 2024; He et al., 2026). Impairment of these interconnected regulatory processes contributes to declining eggshell quality, reduced albumen functionality, diminished yolk stability, and progressive deterioration of overall product integrity during later stages of production. Consequently, reproductive aging should be regarded as a multifactorial biological process that simultaneously influences numerous pathways governing egg quality.

Storage conditions after egg laying can significantly impact quality due to progressive physicochemical and biochemical changes affecting external and internal characteristics. These include carbon dioxide loss, albumin alkalization, moisture redistribution, protein structural modifications, lipid oxidation, and degradation of functional biomolecules. Together, they reduce albumen viscosity, yolk stability, sensory quality, and overall freshness (Adriaensen et al., 2022; Biesek, 2023; Dai et al., 2025). The extent of quality deterioration depends on storage duration, temperature, humidity, eggshell integrity, and the egg’s condition when laid. This underscores the importance of pre- and post-lay factors in determining final product quality.

**Fig. 2:**
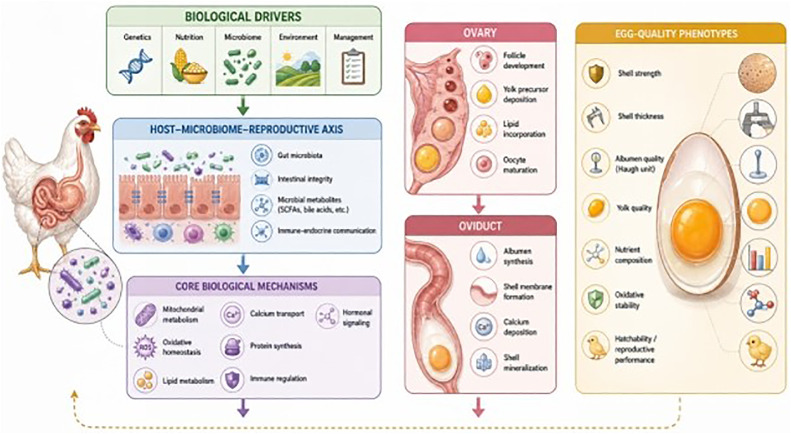
**Biological mechanisms regulating poultry egg quality:** Genetic background, nutrition, microbiome composition, physiological status, and environmental conditions influence egg quality through interconnected host–microbiome–reproductive networks. Microbial metabolites, immune signaling, endocrine regulation, mitochondrial bioenergetics, oxidative homeostasis, nutrient metabolism, calcium and mineral transport, and cellular stress responses collectively regulate ovarian follicular development, yolk deposition, albumen synthesis, eggshell membrane formation, and eggshell biomineralization. These integrated biological processes determine major egg-quality phenotypes, including shell strength, shell thickness, albumen functionality, yolk composition, nutrient value, storage stability, and overall reproductive performance.=.

Integrated transcriptomic, proteomic, metabolomic, lipidomic, epigenomic, and microbiome investigations have substantially expanded understanding of the molecular basis of egg-quality variation (Cui et al., 2021; 2025; Dai et al., 2022; Gao et al., 2025b; Guo et al., 2024b). Current evidence highlights four major biological domains governing outcomes: (i) eggshell biomineralization and calcium homeostasis; (ii) albumen synthesis, secretion, and protein stability; (iii) yolk lipid metabolism, nutrient deposition, and oxidative regulation; and (iv) microbiome-associated metabolic and physiological interactions that influence reproductive performance and nutrient utilization. Multi-omics studies increasingly reveal extensive cross-regulation among these domains, indicating that alterations within one biological system frequently influence multiple downstream quality traits and physiological processes (Fig. 2).

The strongest evidence pertains to eggshell biomineralization and calcium regulatory pathways. Studies employing physiological, molecular, and omics approaches have identified critical transporters, matrix proteins, ion-regulatory mechanisms, and crystal-growth processes that govern shell formation (Cui et al., 2021; 2025; Feng et al., 2023; Fu et al., 2024). Moderate but rapidly expanding evidence supports albumen synthesis, secretory regulation, and yolk deposition pathways via transcriptomic, proteomic, and metabolomic analyses (Dai et al., 2022; Gao et al., 2025b). In contrast, microbiome-mediated influences are an emerging area of investigation. While there is increasing evidence of interactions among gut microbial ecology, nutrient metabolism, reproductive physiology, immune regulation, and egg quality, many of these associations are still correlative. Further research is needed, including controlled intervention studies, longitudinal validation, mechanistic exploration, and functional experimentation, to establish causal relationships (Ferrocino et al., 2023; Gao et al., 2025b; García-Muñoz et al., 2024;).

Current evidence supports the view that egg quality is an emergent systems-level phenotype arising from coordinated interactions among molecular regulation, reproductive physiology, metabolic homeostasis, microbial ecology, environmental influences, and adaptive biological responses. Taken together, this perspective provides an essential foundation for the development of predictive biomarkers, precision-management strategies, and integrative frameworks aimed at improving egg quality across diverse production environments.

### Predictive Multi-Omics Models

Predictive multi-omics approaches are increasingly being used to identify molecular, physiological, metabolic, and microbial signatures associated with egg-quality variation before measurable phenotypic deterioration becomes apparent. This enables earlier intervention and more effective precision-management strategies (Fu et al., 2024; Gao et al., 2025b). These approaches provide comprehensive characterization of the biological processes governing eggshell integrity, albumen functionality, yolk composition, oxidative stability, storage resilience, reproductive performance, and overall product quality by integrating transcriptomic, proteomic, metabolomic, lipidomic, epigenomic, microbiome, physiological, environmental, and phenotypic data (Dai et al., 2022; Fu et al., 2024; Gao et al., 2025b; Guo et al., 2024b). Unlike conventional assessments that primarily detect quality defects after phenotypic manifestation, multi-omics strategies capture upstream biological alterations during trait development, enabling earlier risk identification and supporting proactive management interventions.

Accumulating evidence demonstrates that integrative analyses improve characterization of egg-quality traits by capturing interactions among reproductive physiology, calcium metabolism, mitochondrial function, endocrine regulation, oxidative homeostasis, immune competence, and host–microbiome dynamics (Dai et al., 2022; Fu et al., 2024; Gao et al., 2025b; Guo et al., 2024b). These studies indicate that variation in shell quality, albumen functionality, yolk composition, storage stability, and reproductive efficiency arises from coordinated regulation across multiple biological layers rather than from isolated molecular pathways. Consequently, integration across complementary datasets provides a more comprehensive understanding of egg-quality biology than single-omics analyses alone while simultaneously improving opportunities for biomarker discovery and predictive-model development.

Advances in ML, AI, network biology, and computational systems modeling have further expanded the capacity to analyze high-dimensional biological datasets and identify informative biomarkers associated with egg-quality outcomes (Chen et al., 2025b; Conesa and Beck, 2019; Essien et al., 2026; Hasin et al., 2017). Network-based analytical frameworks enhance mechanistic interpretation by linking candidate biomarkers with pathways involved in eggshell biomineralization, calcium transport, ion homeostasis, albumen synthesis, nutrient deposition, oxidative regulation, reproductive aging, and environmental adaptation (Cui et al., 2021; 2025; Feng et al., 2020; Gao et al., 2025b). These approaches facilitate identification of regulatory hubs, biological interaction networks, emergent system properties, and mechanistically informative biomarkers that may support future applications in breeding, nutrition, health management, and precision-production systems.

Longitudinal investigations demonstrate that temporal biological variation throughout the laying cycle substantially influences eggshell strength, shell ultrastructure, albumen functionality, Haugh-unit values, oxidative stability, and age-associated declines in reproductive performance (Chang et al., 2024a,b; Fu et al., 2024; Gu et al., 2021). Progressive alterations in calcium metabolism, epithelial transport capacity, mitochondrial activity, endocrine regulation, antioxidant defenses, and reproductive physiology collectively contribute to age-related changes in egg quality during commercial production (Chang et al., 2024a,b; Fu et al., 2024; Gao et al., 2025b; Gu et al., 2021; He et al., 2026). Therefore, continuous multi-omics monitoring enables characterization of biological trajectories rather than isolated observations, providing opportunities to identify emerging physiological disturbances before irreversible declines in product quality occur. Such approaches also improve understanding of adaptive responses, physiological resilience, and the mechanisms underlying age-related variation in egg quality.

Growing evidence further suggests that combining microbiome profiles, metabolomic signatures, physiological indicators, and environmental measurements may improve assessment of egg-quality responses to nutritional interventions, heat stress, reproductive aging, and production-system variability (Ferrocino et al., 2023; Gao et al., 2025b; Garcia-Mejia et al., 2024). Nevertheless, many microbiome-associated biomarkers remain insufficiently validated, and most reported relationships remain largely correlative. Therefore, controlled intervention studies, longitudinal validation, mechanistic investigation, and functional experimentation will be required to establish causal relationships, improve reproducibility, and determine practical utility within commercial prediction systems.

Most egg quality prediction platforms are still in the experimental or early validation stages, despite substantial progress. Relatively few studies have demonstrated robust performance across diverse genetic backgrounds, management systems, environmental conditions, and commercial production settings (Emam et al., 2024; Fu et al., 2024; Gao et al., 2025b; Table 3). Limitations include small sample sizes, heterogeneous phenotyping methodologies, and incomplete integration of biological datasets, inadequate external validation, limited mechanistic interpretability, and challenges in commercial implementation. Additionally, many predictive models prioritize statistical accuracy, offering limited insight into underlying biological mechanisms and restricting their adoption in breeding, nutritional, and management programs.

Future progress will depend on large-scale, multi-population validation; harmonized phenotyping strategies; standardized data integration pipelines; interpretable analytical frameworks; longitudinal evaluation; rigorous external validation; and comprehensive cost–benefit assessments. Particular emphasis should be placed on developing biomarker panels that integrate molecular, physiological, microbial, and environmental indicators capable of supporting breeding decisions, precision nutrition, health surveillance, and adaptive management under commercial conditions. The integration of genomic prediction, real-time environmental monitoring, AI, and multi-omics may ultimately enable decision-support systems that proactively manage egg quality throughout the production cycle. Ultimately, the greatest value of predictive multi-omics approaches lies not only in their capacity to forecast quality outcomes but also in their ability to reveal actionable biological mechanisms that support the sustainable improvement of egg quality across diverse production environments.

### Precision Nutrition and Environmental Modulation

Precision nutrition has emerged as a promising strategy for improving egg quality by implementing dietary interventions tailored to a chicken's physiological status, molecular biomarkers, microbiome-derived metabolites, genomic data, environmental factors, and production performance indicators (Emam et al., 2024; Fu et al., 2024; Gao et al., 2025b). Unlike conventional feeding programs, which rely on population-average nutrient requirements, precision nutrition aligns supply with the dynamic biological demands throughout the laying cycle. This approach recognizes the complex interactions among nutrition, reproductive physiology, metabolism, microbial ecology, environmental conditions, and genetic background that influence egg quality outcomes, thereby necessitating adaptive, biologically informed management strategies.

Integrated multi-omics investigations demonstrate that nutritional interventions influence numerous biological processes, including eggshell biomineralization, calcium homeostasis, protein synthesis, lipid metabolism, mitochondrial function, oxidative regulation, immune competence, endocrine signaling, and reproductive performance (Feng et al., 2023; Fu et al., 2024; Gao et al., 2025b). Consequently, dietary strategies can affect multiple aspects of egg quality, such as shell strength, thickness, albumen functionality, Haugh unit values, yolk composition, oxidative stability, storage performance, hatchability, and laying efficiency. Increasing evidence suggests that these responses result from coordinated host-microbiome-metabolism interactions rather than isolated nutrient effects. This underscores the importance of systems-based approaches to the nutritional regulation of egg quality.

A growing body of research suggests that organic trace minerals, phytogenic compounds, probiotics, prebiotics, synbiotics, antioxidants, functional amino acids, vitamins and other bioactive feed additives can improve eggshell quality, albumen stability, yolk characteristics, antioxidant capacity, immune function and laying performance (Dong et al., 2022; Emam et al., 2024; Feng et al., 2023; Ghasemi et al., 2025; Guo et al., 2022). These improvements are mediated through coordinated effects on calcium metabolism, intestinal integrity, redox homeostasis, mitochondrial function, nutrient utilisation, endocrine signalling and host–microbiome interactions. This optimises the physiological processes underlying egg formation and quality maintenance (Ferrocino et al., 2023; Fu et al., 2024; Gao et al., 2025b). Overall, the available evidence suggests that nutritional interventions influence egg quality via interconnected physiological, metabolic, microbial and regulatory networks. This emphasises the multifactorial biological mechanisms that govern egg traits in commercial production settings.

However, responses to dietary interventions exhibit substantial biological variability and are influenced by age, reproductive stage, physiological condition, health status, microbiome composition, environmental conditions, genetic background, and management practices (Fu et al., 2024; Gao et al., 2025b; Guo et al., 2024a). For example, reproductive aging alters calcium metabolism, mitochondrial function, endocrine regulation, and nutrient-utilization efficiency, thereby modifying both nutritional requirements and responsiveness to supplementation strategies (Chang et al., 2024a,b; Fu et al., 2024). Consequently, effective nutritional management increasingly requires flock-specific and context-dependent approaches that account for biological variation throughout the production cycle.

Emerging precision-feeding platforms integrate physiological biomarkers, metabolomic profiles, microbiome data, environmental measurements, production phenotypes, and automated monitoring technologies to support adaptive nutritional management under commercial conditions (Doornweerd et al., 2024; Fu et al., 2024; Gao et al., 2025b). Advances in sensor technologies, AI, ML, and digital-agriculture systems enable continuous monitoring of environmental conditions, bird behavior, physiological status, and production performance, creating opportunities for real-time nutritional adjustment and data-driven decision support (Dhungana et al., 2025; Elwakeel, 2025; Essien et al., 2026). Collectively, these approaches have substantial potential to improve resource-use efficiency, environmental sustainability, animal welfare, production resilience, and the consistency of egg-quality outcomes across diverse production systems.

Environmental stressors remain major determinants of egg quality and can substantially influence the effectiveness of nutritional interventions. In particular, heat stress disrupts reproductive physiology, calcium metabolism, oxidative balance, endocrine regulation, nutrient utilization, intestinal integrity, immune function, and mitochondrial activity, thereby contributing to reductions in shell quality, albumen functionality, laying performance, and overall product quality (Emam et al., 2024; Fu et al., 2024; Gao et al., 2025c). Multi-omics investigations demonstrate that it induces coordinated transcriptomic, proteomic, metabolomic, microbiome, and physiological responses involving oxidative stress, inflammation, metabolic dysregulation, endocrine disruption, and cellular stress pathways that collectively impair reproductive performance and egg-quality maintenance. These findings highlight heat stress as a systems-level biological challenge requiring integrated nutritional, physiological, and environmental management strategies.

Despite encouraging progress, high analytical costs, limited biomarker validation, difficulties associated with integrating heterogeneous datasets, computational-infrastructure requirements, workforce-training demands, data-management complexity, and uncertainty regarding economic returns under commercial conditions constrain widespread implementation (Doornweerd et al., 2024; Essien et al., 2026; Goossens et al., 2022). Moreover, many proposed systems remain at the proof-of-concept or early-validation stage, and relatively few studies have demonstrated consistent biological, operational, and economic benefits across independent commercial populations and production environments.

Future research should prioritize large-scale commercial validation, longitudinal assessment across complete laying cycles, harmonized analytical workflows, standardized biomarker-evaluation protocols, interpretable frameworks, rigorous external validation, and comprehensive cost–benefit analyses. Particular emphasis should be placed on integrating nutritional, physiological, microbial, environmental, and genetic information into unified decision-support platforms capable of delivering actionable recommendations under real-world production conditions. Ultimately, successful implementation will depend on the development of adaptive precision-production systems that translate biological knowledge into practical management strategies capable of consistently improving egg quality, reproductive performance, sustainability, and economic efficiency across diverse commercial environments.

### Translational perspective for predictive egg-quality systems

Advances in multi-omics technologies have substantially expanded understanding of the biological determinants of eggshell integrity, albumen functionality, yolk composition, oxidative stability, storage performance, reproductive efficiency, and overall egg quality throughout the laying cycle (Dai et al., 2022; 2025; Feng et al., 2020; Fu et al., 2024; Gao et al., 2025b). Integrated analyses encompassing transcriptomic, proteomic, metabolomic, lipidomic, epigenomic, microbiome, physiological, and environmental datasets have revealed regulatory mechanisms governing egg formation, biomineralisation, nutrient deposition, reproductive ageing, oxidative regulation, and environmental adaptation (Cui et al., 2021; 2025; Fu et al., 2024; Gao et al., 2025b; Guo et al., 2024b). These advances have enabled the identification of candidate biomarkers, molecular signatures, and physiological indicators that provide a scientific foundation for practical egg-quality monitoring, prediction, and management applications.

The primary challenge in translational research is no longer the generation of biological data but rather the conversion of complex, multidimensional information into scalable, cost-effective, interpretable, and operationally useful tools for commercial egg production (Doornweerd et al., 2024; Elwakeel, 2025; Essien et al., 2026). Consequently, research increasingly focuses on developing streamlined biomarker panels, interpretable predictive models, sensor-enabled monitoring systems, and integrated decision support platforms that translate molecular and physiological measurements into actionable management recommendations (Doornweerd et al., 2024; Elwakeel, 2025; Fu et al., 2024). These technologies can be applied to breeding programs, nutritional management, reproductive monitoring, flock health assessment, environmental control, quality assurance, and production risk mitigation.

Recent advances in AI, ML, network biology, precision agriculture, and digital livestock technologies further enhance opportunities for commercial implementation by facilitating the integration of heterogeneous biological, environmental, and production datasets (Chen et al., 2025b; Dhungana et al., 2025; Essien et al., 2026). Automated monitoring systems incorporating environmental sensors, behavioral observations, physiological measurements, and production indicators increasingly enable real-time assessment of flock status and early detection of biological disturbances that may compromise egg quality (Doornweerd et al., 2024; Elwakeel, 2025). Integration of these technologies with validated biomarker panels and predictive analytical frameworks may support proactive management strategies that improve production consistency, enhance biological resilience, and reduce quality-associated losses.

Among prospective applications, one of the most practical and commercially feasible pathways toward deployment is targeted biomarker panels. Integrating indicators of calcium metabolism, eggshell biomineralization, oxidative status, reproductive physiology, mitochondrial function, immune competence, and microbial activity may provide biologically informative assessments while avoiding the cost and complexity associated with large-scale discovery-oriented omics platforms (Fu et al., 2024; Gao et al., 2025b; Guo et al., 2024b). Such systems could support early detection of physiological disturbances, evaluation of intervention efficacy, and optimization of management decisions throughout the laying cycle. However, successful implementation will require rigorous validation to ensure robustness, reproducibility, biological relevance, and reliable performance across diverse genetic backgrounds, production systems, and environmental conditions.

Despite substantial progress, several barriers continue to limit widespread adoption. These include restricted external validation, heterogeneity in phenotyping methodologies, incomplete integration of biological datasets, analytical costs, computational-infrastructure requirements, workforce-training demands, operational complexity, and uncertainty regarding economic returns under commercial conditions (Doornweerd et al., 2024; Essien et al., 2026; Goossens et al., 2022). Furthermore, many proposed technologies remain at the proof-of-concept or early-validation stage, and relatively few have demonstrated consistent biological, operational, and economic benefits under large-scale production settings. Limited mechanistic interpretability and inconsistent reproducibility across independent populations further constrain confidence in many emerging predictive platforms.

Future progress will depend on large-scale multi-population validation, standardized phenotyping strategies, harmonized analytical workflows, implementation research, rigorous external validation, and comprehensive economic assessments that evaluate both biological effectiveness and commercial feasibility. Priority should be given to the development of affordable biomarker assays, integration of real-time physiological and environmental monitoring, enhancement of mechanistic transparency, and establishment of robust decision-support systems suitable for diverse production environments. Longitudinal investigations spanning multiple laying cycles, genetic backgrounds, and environmental conditions will be particularly important for evaluating long-term reliability, scalability, and operational value.

Ultimately, the successful translation of predictive egg-quality systems will depend on the integration of multi-omics biology, precision nutrition, reproductive physiology, environmental monitoring, and advanced analytics into unified management frameworks. Such systems have the potential to transform production from a reactive quality-control paradigm into a proactive, predictive, and adaptive management model capable of continuously optimizing egg quality, flock performance, sustainability, and economic efficiency across commercial production systems (Fig. 7).

## Biological architecture underlying poultry quality across systems

### Mitochondrial bioenergetics, redox homeostasis, and host–microbiome interactions

Mitochondria play a crucial role in influencing the health, productivity, physiological resilience, and product quality of poultry. They achieve this by regulating cellular energy metabolism, calcium homeostasis, redox signaling, apoptosis, biosynthesis, and adaptive stress responses (Abasht et al., 2016; Bottje, 2019; Chen et al., 2022; Ding et al., 2026; Greene et al., 2025). Mitochondria impact nutrient use efficiency, muscle development, reproductive performance, eggshell biomineralization, immune competence, thermoregulation, and adaptation to environmental challenges through oxidative phosphorylation and metabolic regulation (Bottje, 2019; Chen et al., 2022; Fu et al., 2024; Gao et al., 2024; García-Muñoz et al., 2024; Greene et al., 2025). Consequently, mitochondrial performance is an increasingly important determinant of biological function across poultry production systems, mediating the link between genetics, nutrition, physiology, and environmental adaptation.

Integrated transcriptomic, proteomic, metabolomic, and physiological studies have linked mitochondrial dysfunction to various economically significant traits. These include wooden breast syndrome, white striping, oxidative deterioration of meat quality, reproductive aging, reduced eggshell quality, declining albumen quality, impaired immune function, diminished stress tolerance, and decreased production efficiency (Abasht et al., 2016; Chen et al., 2022; Fu et al., 2024; Gao et al., 2025b, 2026; Greene et al., 2025). Disruptions in tissue integrity, physiological regulation, and product quality have been attributed to alterations in mitochondrial respiration, ATP production, calcium regulation, reactive oxygen species generation, biogenesis, and metabolic flexibility. These findings suggest that mitochondrial bioenergetics serves as a critical interface through which genetic, nutritional, microbial, and environmental factors influence poultry performance and product quality.

The maintenance of cellular redox homeostasis is closely linked to mitochondrial biology. Redox regulation influences metabolism, immune competence, signal transduction, tissue repair, cellular communication, and adaptive physiological responses throughout the organism (Akbarian et al., 2016; Calik et al., 2022; Chen et al., 2024). Under normal physiological conditions, reactive oxygen species function as essential signaling molecules involved in communication, metabolic adaptation, and stress responses. However, excessive production or inadequate antioxidant defenses can result in oxidative stress characterized by lipid peroxidation, protein oxidation, DNA damage, inflammation, mitochondrial dysfunction, and cellular injury (Akbarian et al., 2016; Bi et al., 2024; Chen et al., 2022). Such disturbances have been associated with deterioration of meat quality, eggshell weakening, albumen degradation, reproductive dysfunction, impaired feed efficiency, compromised immunity, and reduced resilience to environmental stressors (Abd El-Samee et al., 2019; Chen et al., 2022; 2024; Gao et al., 2025b).

Mitochondrial function and redox homeostasis are intrinsically interconnected. Mitochondria are both major sources and major targets of reactive oxygen species, creating bidirectional relationships between energy metabolism and oxidative balance (Bottje, 2019; Greene et al., 2025). Dysfunction can amplify oxidative stress through excessive generation, whereas sustained oxidative damage further impairs performance, creating self-reinforcing feedback loops that contribute to physiological dysfunction, reduced adaptive capacity, and progressive quality deterioration. This reciprocal relationship highlights the importance of maintaining mitochondrial integrity and redox balance as complementary targets for improving poultry health, resilience, and product quality.

The intestinal microbiome constitutes a third major component of this regulatory architecture. Gut microbial communities contribute to nutrient digestion, metabolite production, immune-system development, intestinal-barrier integrity, endocrine signaling, and host metabolic regulation (Aruwa et al., 2021; Calik et al., 2022; He et al., 2023). Microbiome composition and activity have been associated with feed efficiency, growth performance, oxidative status, thermotolerance, laying performance, nutrient utilization, intestinal health, reproductive physiology, and product-quality traits in both broilers and laying hens (Bernard et al., 2024; Calik et al., 2022; Guo et al., 2022; Zhou et al., 2023). Microbial metabolites, particularly SCFAs and other bioactive compounds, influence metabolic homeostasis, immune regulation, inflammatory control, mitochondrial activity, and nutrient partitioning, thereby affecting numerous downstream physiological and production-related phenotypes.

**Fig. 3:**
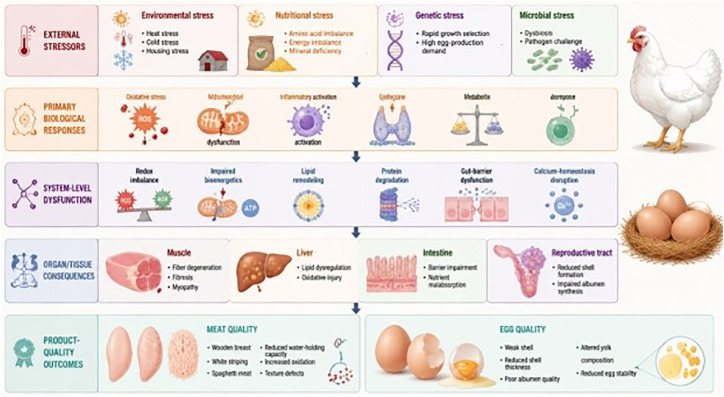
**Mechanistic pathways linking biological and environmental stressors to poultry meat and egg quality deterioration:** Environmental challenges, nutritional imbalance, selective breeding pressure, and gut microbial dysbiosis initiate interconnected oxidative, mitochondrial bioenergetic, inflammatory, endocrine, metabolic, and immune responses. These perturbations disrupt cellular homeostasis and promote tissue-specific dysfunction in skeletal muscle, liver, intestine, ovary, and oviduct. Progressive impairment of bioenergetics, redox regulation, nutrient utilization, and reproductive physiology contributes to reduced meat quality, growth-related myopathies, compromised eggshell formation, diminished albumen quality, altered yolk composition, and reduced overall product quality and performance. Solid arrows indicate well-established mechanisms supported by experimental poultry evidence, whereas dashed arrows represent emerging pathways requiring further validation.

Increasing evidence indicates extensive cross-regulation among microbial ecology, mitochondrial function, and redox homeostasis. Microbiome-derived metabolites influence mitochondrial metabolism, oxidative balance, immune activity, endocrine signaling, and nutrient utilization, whereas host metabolic status shapes microbial composition through reciprocal host–microbiome interactions (Calik et al., 2022; Garcia-Mejia et al., 2024; He et al., 2023). These multidirectional interactions establish interconnected regulatory networks that influence physiological adaptation, environmental resilience, and product-quality outcomes across poultry production systems (Fig. 3). Collectively, current evidence supports the concept of a microbiome–mitochondria–redox axis as a fundamental systems-level framework governing poultry health, productivity, adaptation, and quality.

From a systems-biology perspective, this microbiome–mitochondria–redox axis functions as a shared regulatory hub connecting molecular regulation, nutrient metabolism, immune competence, environmental adaptation, and production performance. Disturbances within any component of the axis can propagate across multiple biological levels, generating cascading effects on growth, reproduction, stress resilience, and product quality. Conversely, interventions targeting microbial ecology, mitochondrial efficiency, or redox balance may simultaneously influence multiple economically important traits, highlighting the translational value of integrated biological approaches.

Although evidence supporting these relationships continues to expand, many microbiome-associated findings remain primarily correlative. The interpretation is complicated by variation among production systems, environmental conditions, genetic backgrounds, and analytical methodologies (Haas et al., 2023; Lan et al., 2025). Future progress will require controlled intervention studies, longitudinal investigations, functional validation, causal-inference methodologies, and integrative modeling frameworks capable of resolving complex interactions among host physiology, microbial ecology, mitochondrial function, and environmental influences. Such efforts will be essential for translating systems-level biological knowledge into robust predictive tools and practical management strategies for improving poultry health, resilience, and product quality.

### Endocrine–immune regulation of poultry phenotypes

Endocrine–immune interactions constitute a highly integrated regulatory network that coordinates growth, reproduction, metabolism, stress responses, tissue development, immune competence, and physiological homeostasis in poultry (Ahmed-Farid et al., 2021; Elokil et al., 2024; Emami et al., 2021). The endocrine and immune systems communicate continuously via bidirectional signaling mechanisms, enabling birds to allocate resources efficiently, maintain physiological stability, and adapt to changing environmental and biological demands (Emami et al., 2021; Calik et al., 2022; Biabani et al., 2024; Guo et al., 2024b, Guo et al., 2024a). Collectively influencing nutrient partitioning, metabolic regulation, inflammatory processes, reproductive function, and environmental adaptation, they are a key determinant of physiological performance, resilience, and product quality in poultry production systems.

Hormonal signaling pathways regulate growth, reproduction, appetite, nutrient utilization, calcium metabolism, stress adaptation, and energy homeostasis, while immune pathways coordinate host defense, inflammatory regulation, tissue repair, and the maintenance of physiological integrity (Ahmed-Farid et al., 2021; Emami et al., 2021). Increasing evidence indicates that these systems are interconnected through shared regulatory networks involving glucocorticoids, thyroid hormones, reproductive hormones, cytokines, growth factors, and metabolic mediators (Elokil et al., 2024; García-Muñoz et al., 2024). Consequently, alterations in endocrine activity often affect immune competence, while activation can modify hormonal regulation, metabolism, nutrient allocation, and overall physiological performance. This reciprocal communication enables coordinated responses to nutritional challenges, pathogen exposure, reproductive demands, and environmental stressors.

Further investigations that integrated transcriptomic, proteomic, metabolomic, microbiome, and physiological data indicate that endocrine–immune regulation operates within broader metabolic, oxidative, mitochondrial, and host–microbial networks that collectively shape poultry phenotypes (Bernard et al., 2024; Calik et al., 2022; Chen et al., 2022; Emami et al., 2021). Endocrine and immune signals influence the allocation of biological resources among competing functions, including growth, reproduction, maintenance, adaptation, and host defense. Through these interactions, endocrine–immune regulation affects feed efficiency, muscle development, eggshell formation, reproductive performance, stress tolerance, disease resistance, and product quality. These observations support the concept that many economically important traits arise from coordinated regulation across interconnected physiological systems rather than isolated molecular pathways.

Environmental stressors are among the most significant modulators of endocrine–immune function. Factors such as heat stress, nutritional imbalances, pathogen challenges, housing conditions, management-related stressors, and other perturbations can alter endocrine signaling, immune responsiveness, inflammatory status, oxidative balance, mitochondrial function, and metabolic regulation. These changes can influence the expression of genetic potential and production outcomes (Akbarian et al., 2016; Chaudhary and Mishra, 2024; Emami et al., 2021). Chronic or severe stress can lead to prolonged activation of stress response pathways, particularly neuroendocrine and inflammatory mechanisms. This may result in impaired immune competence, reduced reproductive efficiency, altered nutrient utilization, and disruption of physiological homeostasis. Such disturbances have been associated with declines in production performance, health status, environmental resilience, and product quality. Therefore, endocrine–immune regulation represents a critical biological interface through which environmental conditions influence adaptive capacity and phenotypic expression.

Recent multi-omics studies have further demonstrated that endocrine–immune networks contribute to variations in meat and egg quality through shared mechanisms involving metabolic regulation, oxidative balance, mitochondrial activity, calcium homeostasis, inflammatory signaling, and cellular stress responses (Cui et al., 2024; Ding et al., 2025; Fu et al., 2024; Gao et al., 2025b). For example, in broiler chickens, dysregulation of endocrine and inflammatory pathways has been associated with growth-related myopathies, oxidative deterioration, metabolic imbalance, impaired muscle integrity, and reduced physiological resilience (Abasht et al., 2016; Greene et al., 2025). In laying hens, endocrine–immune interactions influence follicular development, reproductive aging, eggshell biomineralization, calcium utilization, oxidative stability, and the maintenance of egg quality throughout the laying cycle (Feng et al., 2023; Fu et al., 2024; Gao et al., 2025b). Together, these findings suggest that coordination acts as a universal regulatory mechanism linking various poultry phenotypes across different production systems.

Emerging evidence also highlights extensive interactions among endocrine signaling, immune regulation, and intestinal microbial ecology. Microbial metabolites and signaling molecules can influence endocrine activity, immune responsiveness, metabolic regulation, and stress adaptation, whereas hormonal and immune factors shape microbial composition, intestinal function, and host–microbiome communication (Bernard et al., 2024; Calik et al., 2022; Garcia-Mejia et al., 2024). These reciprocal relationships contribute to the regulation of nutrient utilization, inflammatory balance, reproductive physiology, metabolic homeostasis, and environmental adaptation, further reinforcing the integrated nature of poultry biological systems. Increasingly, the microbiome is recognized as an active participant in endocrine–immune regulation rather than a passive component of host physiology.

From a systems biology perspective, endocrine–immune regulation functions as a central adaptive control network that integrates internal physiological signals with external environmental inputs. This network helps maintain biological stability while enabling adaptive responses to changing conditions through continuous coordination of metabolic, reproductive, immune, and stress-response pathways. Therefore, disturbances within endocrine–immune regulation can propagate across multiple physiological systems, generating cascading effects on growth, reproduction, health, resilience, and product quality. Conversely, interventions that improve endocrine balance, immune competence, or stress resilience may simultaneously enhance multiple economically important traits, highlighting the strategic importance of this regulatory axis for precision poultry production.

Despite substantial progress, many mechanistic relationships among endocrine signaling, immune function, microbiome dynamics, metabolism, and environmental adaptation remain incompletely understood. Future advances will require longitudinal investigations, controlled intervention studies, functional validation, causal-inference methodologies, and integrative multi-omics analyses capable of resolving dynamic interactions across biological scales. Such efforts will be essential for developing predictive biomarkers, mechanistic models, and evidence-based management strategies that improve poultry health, adaptive capacity, production efficiency, and product quality under commercial conditions.

### Adaptive systems framework of poultry quality

Evidence from nutritional, environmental, microbiome-targeted, genetic, and management interventions consistently demonstrates that biological perturbations rarely affect isolated traits. Instead, such interventions induce coordinated changes in metabolism, immune competence, endocrine regulation, mitochondrial function, oxidative balance, behavior, physiological resilience, productivity, welfare, and product quality (Brugaletta et al., 2023a,b; Calik et al., 2022; Emami et al., 2021; Guo et al., 2024a; He et al., 2023; Mancinelli et al., 2023). These observations indicate that poultry-product quality emerges from the collective behavior of interacting systems rather than from individual molecular pathways, isolated biomarkers, or single physiological processes (Beauclercq et al., 2017; Berri et al., 2019; Chen et al., 2022; Cui et al., 2024; Ding et al., 2025). Consequently, explaining variation in meat and egg quality requires an integrative framework capable of linking biological interactions across multiple levels of organization and translating complex mechanistic relationships into coherent principles.

We propose the Adaptive Systems Theory of Poultry Quality (ASTPQ) as a conceptual systems-biology framework developed from the converging experimental and translational evidence synthesized throughout this review. Rather than representing a fully validated biological theory, ASTPQ provides an integrative conceptual architecture that unifies genetic, molecular, physiological, metabolic, microbiome, nutritional, environmental, and management determinants of poultry product quality. Within this framework, adaptive regulatory hubs including mitochondrial bioenergetics, oxidative homeostasis, endocrine–immune coordination, host–microbiome interactions, metabolic regulation, calcium homeostasis, and environmental-response pathways are proposed on the basis of convergent biological evidence, mechanistic plausibility, and repeated associations across independent studies rather than definitive experimental demonstration of causal network centrality. Accordingly, ASTPQ is intended to organize current biological knowledge, generate experimentally testable hypotheses, and guide future mechanistic, predictive, and translational research while remaining open to refinement as additional evidence becomes available.

A central proposition of ASTPQ is that variation in product quality reflects differences in adaptive-system capacity. It encompasses robustness, resilience, phenotypic flexibility, recovery potential, physiological stability, metabolic efficiency, and responsiveness to environmental change. Birds possessing greater adaptive capacity are hypothesized to maintain functional homeostasis more effectively during nutritional, environmental, infectious, reproductive, or metabolic challenges, thereby sustaining superior productivity, welfare, health status, and product-quality outcomes (Biabani et al., 2024; Calik et al., 2022; Emami et al., 2021; Guo et al., 2024a; Haas et al., 2023; He et al., 2023; Mancinelli et al., 2023). Conversely, reduced adaptive capacity may increase susceptibility to physiological dysregulation, oxidative stress, metabolic dysfunction, reproductive decline, impaired resilience, and deterioration of meat or egg quality.

Within ASTPQ, several interconnected systems are proposed as principal adaptive regulatory hubs based on their recurrent association with multiple quality-related biological processes. These include mitochondrial bioenergetics, redox homeostasis, endocrine–immune coordination, calcium regulation, host–microbiome interactions, metabolic regulation, and environmental-response pathways. Although convergent evidence supports their biological importance, direct experimental validation of their regulatory centrality remains incomplete. Multi-omics investigations increasingly demonstrate that these systems influence multiple phenotypes simultaneously, including growth performance, feed efficiency, muscle integrity, reproductive function, eggshell biomineralization, oxidative stability, disease resistance, stress tolerance, and environmental resilience (Cui et al., 2024; Ding et al., 2026; Fu et al., 2024; Gao et al., 2025a,b; Greene et al., 2025). Therefore, ASTPQ proposes that many quality-associated traits share a common adaptive architecture while retaining specialized regulatory pathways that contribute to phenotype-specific variation. In this context, product quality represents the integrated expression of both shared mechanisms and trait-specific biological processes.

Importantly, ASTPQ is not intended to replace established mechanistic explanations of poultry biology. Rather, it serves as an integrative framework for organizing evidence across disciplines and levels of biological organization. The theory proposes that genomic variation, epigenetic regulation, molecular signaling, microbiome dynamics, nutritional inputs, physiological responses, management practices, and environmental conditions interact through adaptive regulatory networks that collectively determine product-quality outcomes. This perspective enables previously disconnected areas of poultry research to be interpreted within a common systems-biology framework while preserving mechanistic specificity.

ASTPQ also generates several testable hypotheses. First, multidimensional indicators of adaptive-system function are expected to explain a greater proportion of biological variation in poultry quality than individual biomarkers evaluated independently. Second, integrated measures of mitochondrial performance, oxidative balance, endocrine–immune status, microbiome functionality, calcium homeostasis, and physiological resilience are predicted to provide earlier and more accurate predictions of quality outcomes than conventional phenotypic indicators alone. Third, interventions that enhance adaptive-system capacity should produce coordinated improvements across production, welfare, health, resilience, and product-quality traits. Emerging evidence from integrated genomic, physiological, and multi-omics investigations supports these predictions by demonstrating improved biological interpretation, predictive performance, and decision-support capability when complementary dimensions are evaluated collectively rather than independently (Ballard et al., 2024; Cui et al., 2024; Ding et al., 2026; Essien et al., 2026; Fu et al., 2022; Gao et al., 2025c, Gao et al., 2025b, Gao et al., 2025a; Wolc et al., 2016).

The framework also establishes a conceptual foundation for developing next-generation predictive poultry systems. Technological advances in areas such as multi-omics integration, precision phenotyping, environmental sensing, wearable monitoring technologies, digital agriculture, systems modeling, network biology, and explainable artificial intelligence (**XAI**) increasingly enable the real-time characterization of adaptive biological processes (Cui et al., 2024; Dhungana et al., 2025; Ding et al., 2025; Elwakeel, 2025; Essien et al., 2026). Integrating these technologies within the ASTPQ framework could facilitate the development of precision systems that are biologically interpretable, predictive, adaptive, and commercially viable. Such systems would be capable of identifying vulnerabilities, forecasting quality deterioration, and guiding intervention strategies before overt phenotypic manifestations occur.

From a translational perspective, ASTPQ provides a scientific foundation for adaptive management strategies, integrated biomarker panels, resilience-oriented breeding programs, precision-feeding systems, environmental-response frameworks, and predictive decision-support platforms. Such applications may facilitate simultaneous improvements in productivity, animal welfare, environmental sustainability, resource-use efficiency, health status, and product quality while strengthening the biological robustness of poultry production systems. Consequently, ASTPQ offers a pathway for translating systems-level biological knowledge into practical tools that support evidence-based decision-making under commercial conditions.

ASTPQ establishes a conceptual bridge between mechanistic biology and predictive production science, beyond its immediate applications. The framework provides a structure for integrating discovery-oriented research with translational implementation by linking molecular regulation with organismal performance and environmental adaptation. This integration may help accelerate biomarker development, improve causal inference, enhance predictive accuracy, and support the design of intervention strategies that are both biologically informed and commercially relevant.

Ultimately, ASTPQ proposes that poultry quality is an emergent property of adaptive biological systems operating across molecular, cellular, physiological, microbial, organismal, management, and environmental scales. Through continuous interactions among these interconnected systems, birds dynamically regulate resource allocation, maintain homeostasis, respond to perturbations, and preserve functional performance under changing conditions. By integrating evidence across these domains, ASTPQ establishes a foundation for hypothesis-driven research, mechanistically informed biomarker discovery, causal systems modeling, and next-generation precision poultry-production strategies capable of improving productivity, welfare, sustainability, resilience, and product quality within increasingly complex environments (Fig. 5; Table 12).

## Translational applications for poultry meat and egg quality

The primary goal of modern poultry systems biology is to translate mechanistic biological knowledge into evidence-based strategies that improve the quality of meat and eggs. These include informed breeding, precise nutrition, health management, optimized environments, and data-driven production systems (Cui et al., 2024; Dai et al., 2022; Ding et al., 2025; Fu et al., 2024; Gao et al., 2025a). Significant advances have been made in multi-omics technologies, systems biology, precision phenotyping, digital agriculture, and computational analytics, deepening our understanding of the molecular and physiological mechanisms that govern product quality. However, a major challenge that remains is converting these discoveries into scalable, economically sustainable, and operationally practical technologies that support decision-making across diverse commercial production systems.

Current evidence identifies precision nutrition, microbiome-informed management, genomic selection, sensor-enabled monitoring, and digital decision-support platforms as the principal translational outcomes of poultry systems biology (Chu et al., 2019; Dai et al., 2022; Doornweerd et al., 2024; Elwakeel, 2025; Essien et al., 2026; Gao et al., 2025a). Among these approaches, precision nutrition and microbiome-directed interventions currently possess the strongest experimental and commercial evidence for improving poultry-product quality, whereas genomic selection and digital monitoring technologies are advancing rapidly toward broader industry adoption. Concurrent developments in AI, ML, computer vision, automated environmental control, and real-time sensing increasingly enable continuous assessment of biological performance and production environments, thereby supporting predictive and adaptive management.

Despite these advances, translational technologies differ substantially in scientific maturity, validation status, implementation readiness, infrastructure requirements, economic feasibility, and scalability (Chu et al., 2019; Doornweerd et al., 2024; Elwakeel, 2025; Essien et al., 2026). Consequently, further research is required to bridge the gap between experimental discovery and routine commercial application.

### Precision nutrition and quality optimization

Precision nutrition represents one of the most mature and commercially relevant applications of poultry systems biology. It translates mechanistic biological knowledge into targeted nutritional strategies that optimize productivity, health, physiological resilience, sustainability, and meat and egg quality (Gao et al., 2017; Ghasemi et al., 2025; Guo et al., 2022). Unlike conventional feeding programs based primarily on population-average nutrient requirements, precision nutrition aligns nutrient supply with dynamic biological demands by integrating physiological status, metabolic condition, microbiome composition, environmental influences, genetic background, and production objectives.

**Fig. 4:**
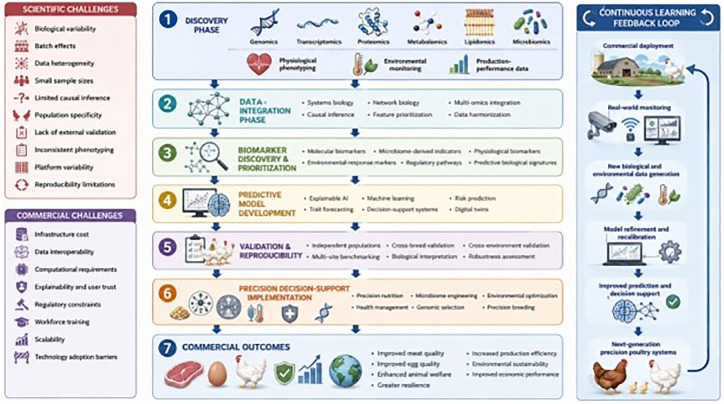
**Translational pipeline for implementation of precision poultry systems:** Multi-omics, phenotypic, physiological, and environmental datasets are integrated through systems biology, network-analysis, and causal inference frameworks to identify biologically relevant pathways, biomarkers, and predictive signatures. These features support the development of XAI models and digital-twin approaches for trait prediction and decision-support systems. Rigorous validation across independent populations, production systems, and environmental conditions is required prior to deployment. Validated models inform precision nutrition, microbiome engineering, environmental management, health monitoring, and genomic-selection strategies. Continuous feedback from commercial implementation enables iterative model refinement and adaptive optimization, thereby improving product quality, animal welfare, production efficiency, sustainability, and economic performance.

Modern precision-feeding systems increasingly integrate molecular, physiological, environmental, and production-performance data to improve nutrient-use efficiency, feed conversion, growth, reproductive performance, flock health, and product quality through biologically informed nutritional management (Bernard et al., 2024; Ghasemi et al., 2025; Guo et al., 2022; Fig. 4). This systems-oriented framework recognizes that nutritional requirements arise from interactions among genetics, metabolism, immune function, microbial ecology, physiological adaptation, and environmental conditions rather than remaining fixed throughout production. Consequently, nutritional interventions influence multiple interconnected biological processes, generating coordinated improvements in performance and product quality.

**Fig. 5:**
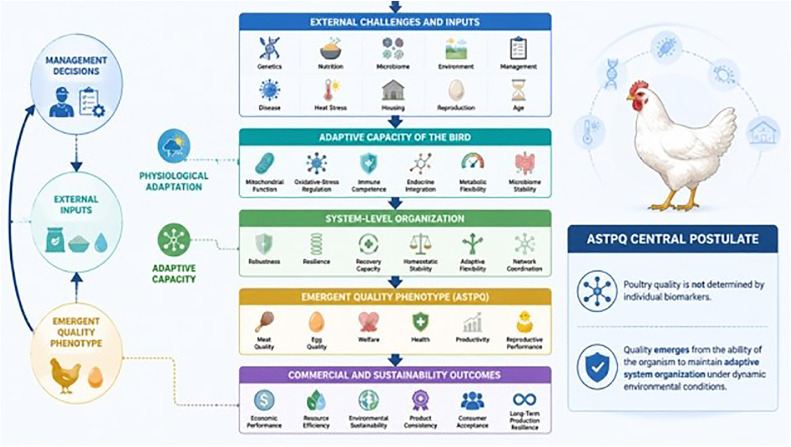
**Adaptive Systems Theory of Poultry Quality (ASTPQ):** A conceptual systems-biology framework for predictive poultry quality: External environmental and biological stressors influence poultry performance through effects on adaptive capacity, including mitochondrial function, oxidative regulation, immune competence, endocrine integration, metabolic flexibility, and microbiome stability. These adaptive processes collectively determine system-level properties such as robustness, resilience, recovery capacity, and homeostatic stability. ASTPQ is proposed as a conceptual framework that integrates current evidence across multiple biological scales and generates testable hypotheses regarding the determinants of poultry product quality. Within this framework, meat quality, egg quality, welfare, health, productivity, and reproductive performance emerge from system organization and adaptive capacity rather than being determined solely by individual molecular determinants. Commercial performance and sustainability outcomes are viewed as downstream manifestations of adaptive-system function, while biological and management feedback loops support continuous adaptation, resilience, and system optimization under changing production conditions.

Consistent experimental evidence demonstrates that amino acids, dietary lipids, antioxidants, trace minerals, vitamins, probiotics, prebiotics, synbiotics, phytogenic compounds, and other bioactive feed additives can enhance meat quality, oxidative stability, fatty acid composition, water-holding capacity, eggshell strength, albumen functionality, yolk quality, intestinal health, immune competence, and physiological resilience (Fu et al., 2024; Gao et al., 2025a; Ghasemi et al., 2025; Guo et al., 2022). Multi-omics studies further reveal that these benefits result from the coordinated regulation of mitochondrial function, redox homeostasis, nutrient metabolism, microbial ecology, endocrine signaling, immune activity, and cellular stress response pathways. This evidence highlights that nutritional interventions exert integrated, systems-level effects rather than isolated, nutrient-specific responses.

Further evidence suggests that a combination of nutritional interventions is more effective than a single nutrient strategy because the quality of meat and eggs depends on interconnected metabolic, physiological, microbial, and molecular networks (Gao et al., 2025a; Ghasemi et al., 2025; Guo et al., 2022). For example, by providing a combination of probiotics, antioxidants, functional amino acids, and trace minerals, it is possible to simultaneously enhance intestinal integrity, microbial metabolism, oxidative regulation, immune competence, and nutrient utilization. This results in improvements in both biological performance and product quality. These findings reinforce the systems biology principle that complex phenotypes are most effectively optimized by coordinating the modulation of multiple pathways.

Precision-feeding platforms are becoming more integrated with environmental sensing, automated data acquisition, physiological biomarkers, microbiome profiles, production indicators, and machine-learning algorithms to support dynamic nutritional management (Doornweerd et al., 2024; Elwakeel, 2025; Essien et al., 2026). These platforms allow for the adaptive adjustment of nutrient supply in response to continuously changing biological and environmental conditions. This improves the efficiency of resource use while enhancing productivity, product quality, animal welfare, and environmental sustainability.

Nevertheless, variability among production systems, inconsistent responses across genetic backgrounds, limited availability of validated biomarkers, analytical costs, data-integration complexity, computational infrastructure requirements, workforce-training demands, and uncertainty regarding long-term economic returns constrain broader implementation (Alves et al., 2024; Bernard et al., 2024; Chu et al., 2019; Doornweerd et al., 2024; Elwakeel, 2025). Moreover, researchers have evaluated many promising nutritional strategies primarily under controlled experimental conditions, with comparatively few demonstrating consistent effectiveness across diverse commercial environments.

Future progress will depend on large-scale commercial validation, multi-environment trials, standardized evaluation protocols, harmonized analytical workflows, implementation research, and comprehensive economic assessments that jointly evaluate biological effectiveness and financial feasibility. Priority should be given to identifying robust biomarkers of nutritional responsiveness, developing biologically interpretable decision-support systems, and establishing cost-effective precision-feeding platforms suitable for routine commercial deployment.

#### Genotype- and phenotype-guided precision feeding

The transition from conventional population-based feeding programs toward precision nutrition requires dietary strategies that account for genetic, physiological, microbial, and environmental sources of biological variation to optimize nutrient utilization, health, production efficiency, and meat and egg quality (Bernard et al., 2024; Chu et al., 2019; Ghasemi et al., 2025). Contemporary precision-feeding systems increasingly integrate genomic information, physiological biomarkers, production phenotypes, gut microbiome characteristics (Table 4), and environmental measurements to align nutrient supply with the dynamic biological requirements of individual birds or management groups. This improves nutritional efficiency and adaptive management throughout the production cycle (Bernard et al., 2024; Chu et al., 2019).

Genetic diversity is a major determinant of digestive capacity, nutrient utilization, feed efficiency, growth, reproductive performance, eggshell biomineralization, stress responsiveness, immune competence, and susceptibility to metabolic disorders (Contriciani et al., 2024; de Verdal et al., 2011). Consequently, birds from different genetic backgrounds exhibit distinct nutritional requirements and variable responses to dietary interventions. This supports genotype-informed nutritional strategies that enhance feed efficiency, production uniformity, product quality, nutrient-use efficiency, and sustainability while minimizing losses (Beauclercq et al., 2017; Contriciani et al., 2024; Fu et al., 2024).

Dynamic phenotypic indicators, such as growth rate, feed intake, body composition, digestive efficiency, metabolic status, physiological biomarkers, microbial characteristics, feeding behavior, laying performance, and egg quality, as well as responses to environmental conditions, complement genomic information by offering real-time evaluations of biological status (Alves et al., 2024; Beauclercq et al., 2018; Bernard et al., 2024; Fu et al., 2024). By integrating these multidimensional phenotypes, dietary formulations can be continuously refined according to developmental stage, production objectives, health status, physiological condition, and environmental challenges. This supports biologically responsive nutritional management throughout production.

Emerging evidence further indicates that microbiome-derived biomarkers and metabolomic signatures can improve nutritional precision by characterizing individual variation in digestive function, microbial metabolic capacity, host metabolism, immune competence, and physiological resilience (Bernard et al., 2024; Brugaletta et al., 2023a, Brugaletta et al., 2023b; Calik et al., 2022). These may also facilitate earlier detection of nutritional imbalance and physiological dysregulation before measurable declines in productivity, animal welfare, or product quality occur, creating opportunities for timely intervention (Bernard et al., 2024; Gautam et al., 2024).

Integrated precision-feeding frameworks combine genomic information, phenotypic measurements, physiological biomarkers, microbiome profiles, and environmental data to stratify birds according to nutritional requirements, adaptive capacity, and predicted production responses (Bernard et al., 2024; Brugaletta et al., 2023a, Brugaletta et al., 2023b; Chu et al., 2019). This multidimensional approach supports targeted nutritional interventions, including amino acid optimization, dietary lipid modification, antioxidant supplementation, precision mineral nutrition, and microbiome-directed feeding strategies, to improve feed efficiency, physiological resilience, meat quality, egg quality, and sustainability-related production outcomes (Bi et al., 2024; Bortoluzzi et al., 2025; Dev et al., 2020; Elkin et al., 2015; Ferrocino et al., 2023; Ghasemi et al., 2020).

Large-scale commercial implementation remains at an early stage, but rapid advances in sensor technologies, automated phenotyping, environmental monitoring, biomarker discovery, AI, and decision-support systems are accelerating the development of commercially deployable precision-feeding platforms (Doornweerd et al., 2024; Elwakeel, 2025; Essien et al., 2026). Future progress will depend on rigorous validation across diverse genetic lines, production systems, and environmental conditions; development of robust and economically practical biomarker panels; and seamless integration of multidimensional biological and environmental information into scalable decision-support frameworks capable of delivering reproducible improvements in product quality, production efficiency, animal welfare, economic performance, and environmental sustainability.

### Microbiome-guided environmental monitoring and adaptive management

Microbiome-directed nutritional interventions, including probiotics, prebiotics, synbiotics, postbiotics, and phytogenic feed additives, have demonstrated considerable potential to enhance nutrient utilization, intestinal barrier integrity, microbial homeostasis, immune competence, antioxidant defenses, and productive performance through modulation of host–microbiome interactions and metabolism (Dev et al., 2020; Elbaz et al., 2023; Ferrocino et al., 2023). Although responses vary according to host genetics, age, dietary composition, management practices, environmental conditions, and baseline microbial communities, accumulating evidence indicates that microbiome-informed management strengthens precision nutrition and flock-health programs by improving digestive efficiency, physiological resilience, disease resistance, animal welfare, and the consistency of meat and egg quality while reducing reliance on antimicrobial interventions (Bernard et al., 2024; Bortoluzzi et al., 2024; Gautam et al., 2024).

Environmental conditions remain major determinants of poultry health, productivity, and product quality. Heat stress, humidity, ventilation, air quality, stocking density, lighting, litter management, and related husbandry factors influence nutrient utilization, growth, reproductive performance, eggshell biomineralization, oxidative balance, immune competence, microbial stability, and the consistency of meat- and egg-quality traits (Ahmed-Farid et al., 2021; Emam et al., 2024; Emami et al., 2021). Therefore, effective environmental management is a fundamental component of precision poultry production.

Recent advances in precision livestock technologies enable real-time assessment of environmental conditions, animal behavior, physiological responses, and production performance. These include environmental sensors, machine-vision systems, wearable and contact-free monitoring devices, automated phenotyping platforms, and AI-assisted decision-support tools (Balthazar et al., 2025; Doornweerd et al., 2024; Elwakeel, 2025; Essien et al., 2026). Integration of these multidimensional data streams with microbiome profiles, phenotypic measurements, and production records facilitates early detection of environmental stress, declining health, nutritional imbalance, and disease risk. This supports timely evidence-based interventions that improve productivity, animal welfare, product quality, and environmental sustainability.

Future advances will depend on the rigorous validation of integrated microbiome–environment monitoring frameworks across a range of genetic backgrounds, production systems, management practices, and climatic conditions. Developing standardized microbiome-derived biomarkers, interoperable sensing technologies, XAI models, and scalable decision support platforms will be crucial in translating precision environmental management into commercially viable strategies that can deliver consistent, reproducible improvements in poultry health, production efficiency, meat and egg quality, economic performance, and environmental sustainability.

### Commercial readiness, precision monitoring, and decision-support systems

Digital agriculture has accelerated the integration of environmental sensing, automated phenotyping, physiological monitoring, genomic information, biomarker profiling, production analytics, and AI into modern poultry production (Chu et al., 2019; Doornweerd et al., 2024; Elwakeel, 2025; Essien et al., 2026) (Table 5). These technologies collectively enable continuous assessment of flock health, welfare, environmental conditions, production performance, and quality-related risk factors, facilitating early detection of production challenges, timely intervention, and evidence-based management throughout the production cycle (Doornweerd et al., 2024; Elwakeel, 2025; Essien et al., 2026).

Among currently available precision-production technologies, genomic selection, precision nutrition, environmental monitoring, automated environmental control, sensor-based management platforms, and targeted biomarker applications (Abd El-Hack et al., 2025; Aggrey et al., 2010; Chu et al., 2019; Elwakeel, 2025). Together, these have demonstrated measurable improvements in feed utilization, production efficiency, environmental resilience, animal welfare, resource-use efficiency, and the consistency of poultry meat and egg quality under commercial conditions (Abd El-Hack et al., 2025; Aggrey et al., 2010; Chu et al., 2019; Elwakeel, 2025).

In contrast, integrated multi-omics platforms, ML methods, and AI–driven analytical frameworks including digital twins, multimodal data fusion, and federated learning architectures remain largely at the developmental, pilot validation, or proof-of-concept stage (Ballard et al., 2024; Essien et al., 2026; Fu et al., 2022; Goossens et al., 2022). Broader implementation is constrained by analytical and computational costs, infrastructure requirements, data interoperability, cybersecurity and governance considerations, workforce expertise, and the limited availability of large-scale, multi-site, commercial validation studies (Ballard et al., 2024; Essien et al., 2026; Goossens et al., 2022). Increasingly sophisticated systems may not always justify their implementation, maintenance, and operational costs, emphasizing the need for rigorous techno-economic evaluations and cost-effectiveness analyses before large-scale deployment (Table 8).

Future progress will require robust external validation across diverse genetic backgrounds, production systems, geographical regions, and environmental conditions. This must be accompanied by interoperable data infrastructures, standardized implementation frameworks, and comprehensive economic, operational, and sustainability assessments. Transparent, biologically interpretable AI models that can integrate genomic, phenotypic, microbiome, environmental, and production data into scalable decision support platforms will also be crucial. Ultimately, successful implementation will depend on translating complex biological and production information into accurate, explainable, economically viable, and operationally practical recommendations that enhance poultry health, production efficiency, animal welfare, and the quality of meat and eggs in a sustainable way across diverse commercial systems.

## Translational and deployment barriers

Despite major advances in poultry genomics, multi-omics technologies, precision nutrition, microbiome science, AI, and predictive analytics, routine commercial adoption remains limited. This translational gap reflects persistent challenges in developing predictive models and decision-support frameworks that are biologically interpretable, reproducible, operationally practical, economically scalable, and robust across diverse genetic backgrounds, production systems, and environmental conditions. Model performance is strongly influenced by genotype × environment × management interactions, biological heterogeneity, temporal physiological variation, inconsistencies in farm-level data acquisition, and differences in husbandry practices and production environments (Chu et al., 2019; de Kinderen et al., 2023; Essien et al., 2026; Goossens et al., 2022).

Increasing molecular complexity alone does not necessarily improve predictive performance. Evidence from poultry genomics, multi-omics integration, and precision-production research demonstrates that prediction accuracy depends primarily on data quality, biological relevance, experimental design, sample size, feature selection, model interpretability, and rigorous external validation rather than the quantity of molecular information incorporated. Under conditions of limited sample size, inadequate external validation, heterogeneous production environments, or poorly harmonized datasets, additional omics layers may increase analytical complexity, computational demands, and the risk of overfitting without proportionate gains in predictive accuracy, robustness, or generalizability (Argelaguet et al., 2018; 2020; Ballard et al., 2024; Goossens et al., 2022).

Microbiome-derived biomarkers present similar translational challenges because microbial community composition and host–microbiome interactions are highly dynamic and influenced by age, dietary composition, disease status, antimicrobial exposure, housing conditions, environmental stressors, and temporal variation (Aruwa and Sabiu, 2024; Bajagai et al., 2024; Bernard et al., 2024; Gautam et al., 2024). Consequently, models that perform well within individual experimental cohorts often exhibit substantially reduced robustness when applied across independent commercial populations differing in genetics, management, geography, or production environment.

Additional barriers include the absence of standardized sampling protocols, limited interoperability among data platforms, inconsistent metadata collection, regulatory and data-governance requirements, high implementation and maintenance costs, shortages of multidisciplinary expertise, and the limited availability of large-scale prospective commercial validation studies. Together, these constraints continue to impede routine integration of multi-omics, microbiome, and AI technologies into precision poultry production (Ballard et al., 2024; Essien et al., 2026; Goossens et al., 2022).

Overall, current evidence indicates that successful translation depends less on increasing molecular complexity than on improving biological interpretability, mechanistic understanding, data standardization, external validation, robustness across diverse production environments, operational scalability, and economic feasibility. Future progress will require parsimonious, mechanistically informed, explainable, and commercially scalable prediction frameworks that integrate genomic, phenotypic, microbiome, environmental, and production information into transparent decision-support systems capable of delivering reproducible, biologically meaningful, and economically sustainable improvements in poultry health, production efficiency, animal welfare, and meat and egg quality.

### Commercial generalizability and validation for deployment

Commercial poultry production is characterized by substantial genetic, nutritional, environmental, microbial, physiological, and management heterogeneity that collectively shapes phenotypic expression and complicates predictive modeling across genotype × environment × management interactions (Bernard et al., 2024; Chu et al., 2019; de Kinderen et al., 2023). Consequently, predictive models developed under controlled experimental conditions frequently lose accuracy when applied across different genetic lines, geographic regions, production systems, housing environments, nutritional programs, and management practices because of biological variability, temporal dynamics, and distributional shifts between training and deployment populations (Bernard et al., 2024; Chu et al., 2019; de Kinderen et al., 2023). Therefore, improving generalizability requires rigorous evaluation across genetically diverse populations, heterogeneous production environments, and large multi-site commercial datasets to ensure reliable out-of-sample prediction and facilitate development of robust decision-support systems for poultry meat and egg quality management (Chu et al., 2019; de Kinderen et al., 2023; Essien et al., 2026; Goossens et al., 2022).

Successful deployment also depends on comprehensive external validation extending beyond discovery cohorts. This is because predictive relationships frequently vary across genetic backgrounds, nutritional strategies, microbial communities, management systems, and production environments, limiting transferability to independent commercial populations (Bernard et al., 2024; Chu et al., 2019; de Kinderen et al., 2023; Gautam et al., 2024). Reproducibility is further challenged by biological and technical variation arising from host genetics, age, health status, environmental exposures, sampling strategies, laboratory methodologies, sequencing platforms, bioinformatics pipelines, and temporal dynamics. All of these factors contribute to heterogeneity in molecular profiles among independent studies (Bernard et al., 2024; Gautam et al., 2024; Goossens et al., 2022). Therefore, distinguishing reproducible biological signals from context-dependent associations is essential before predictive models can be translated into commercial practice.

To strengthen the translational utility of poultry multi-omics and precision-production frameworks, it will be necessary to implement standardized experimental designs, harmonized sampling procedures, interoperable data standards, validated bioinformatics and statistical workflows, transparent reporting, longitudinal multi-environment investigations, prospective multi-site field validation, and independent evaluation across diverse commercial systems. Benchmark datasets and standardized performance metrics will enable objective comparisons of predictive models across independent studies. Together, these advances will improve reproducibility, strengthen external validity, enhance robustness, and accelerate the development of biologically interpretable, operationally scalable, and commercially applicable decision support systems that can consistently improve poultry health, production efficiency, animal welfare, and meat and egg quality under real-world production conditions.

### Economic and infrastructure constraints

Commercial implementation of precision poultry technologies requires substantial investment in sensing networks, automated phenotyping, data acquisition, digital infrastructure, computational resources, and decision-support platforms that support continuous collection, real-time analytics, and evidence-based management (Doornweerd et al., 2024; Elwakeel, 2025; Essien et al., 2026). These requirements increase capital expenditure, operational complexity, data-management demands, and workforce-training needs, particularly in large commercial enterprises. Current evidence indicates that genomic selection, precision nutrition, environmental monitoring, automated environmental control, and sensor-based management represent the most mature and commercially validated technologies for improving production efficiency, animal health, welfare, resource-use efficiency, and meat and egg quality (Aggrey et al., 2010; Chu et al., 2019; Doornweerd et al., 2024).

By comparison, integrated multi-omics frameworks require substantially greater laboratory capacity, analytical expertise, computational infrastructure, and bioinformatics support. They frequently exhibit variable predictive performance across traits, genetic populations, production environments, and management systems. This variability reflects differences in genetic architecture, population structure, genotype × environment interactions, biological heterogeneity, sample size, data quality, and environmental variation documented across poultry studies (Bernard et al., 2024; Chu et al., 2019; Cui et al., 2024; Goossens et al., 2022). Consequently, broader commercial adoption will depend on demonstrating reproducible biological utility, operational feasibility, consistent predictive performance, and measurable economic value.

Among omics-based technologies, genomic selection is the most economically mature application. This is because genotyping costs are distributed across large breeding populations, and cumulative genetic gains are realized over successive generations through continual improvement of breeding-value prediction (Aggrey et al., 2010; Chu et al., 2019) (Table 6). In contrast, transcriptomic, proteomic, metabolomic, and microbiome-based approaches typically necessitate repeated biological sampling, as well as more extensive laboratory processing, analytical expertise, computational infrastructure, and data interpretation. This results in substantially higher per-animal phenotyping costs and greater implementation complexity (Bernard et al., 2024; Cui et al., 2024; Goossens et al., 2022).

The future deployment of integrated multi-omics systems will depend on robust, cost-effective biomarker panels, reduced-feature predictive models, automated analytical workflows, and scalable computational pipelines that deliver measurable advantages over existing genomic-selection and precision-management strategies. A comprehensive techno-economic evaluation, including cost–benefit analysis, return on investment, and operational feasibility, will be equally important to determine whether additional biological complexity generates meaningful commercial value. Ultimately, successful implementation will depend not only on predictive accuracy but also on reproducibility, scalability, affordability, interoperability, operational practicality, and sustained economic return.

### Computational scalability and explainable AI for decision support

**Fig. 6:**
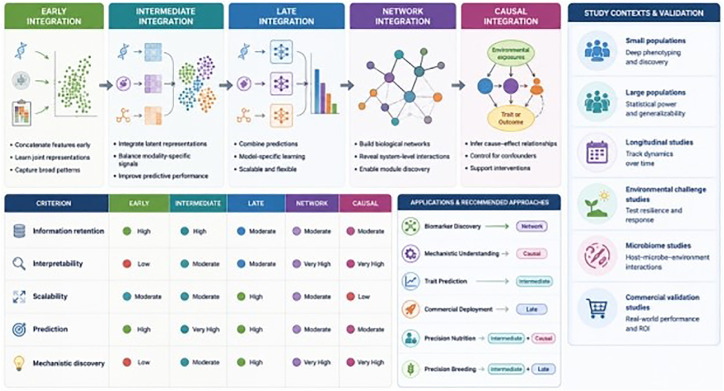
**Comparative multi-omics integration architectures for poultry quality research:** This Fig. compares early integration, intermediate integration, late integration, network-based integration, and causal-inference–driven multi-omics frameworks used in poultry quality research. The comparison highlights key methodological characteristics, including data integration timing, dimensionality handling, model complexity, interpretability, and biological resolution. It further illustrates trade-offs in predictive performance, mechanistic interpretability, computational demand, and scalability across meat quality, egg quality, health, and production trait applications. Validation requirements across populations, environments, and production systems are also contrasted, emphasizing differences in robustness, generalizability, and translational readiness among integration strategies.

Multi-omics integration can be achieved through various analytical strategies, each offering unique advantages depending on the specific biological question, dataset characteristics, and intended application (Argelaguet et al., 2020; Fu et al., 2022). Early integration combines multiple omics layers before model development. While this approach maximizes information capture, it also substantially increases dimensionality, computational demands, and the risk of overfitting. Intermediate integration extracts biologically informative latent features from individual layers prior to integration. This reduces noise, improves computational efficiency, and enhances downstream analysis. In late integration, predictions from separate omics-specific models are combined to improve modularity, flexibility, and interpretability. However, cross-omics interactions may be less comprehensively represented than in fully integrated approaches (Argelaguet et al., 2020; Ballard et al., 2024). Network-based frameworks identify interactions among genes, transcripts, proteins, metabolites, microbial taxa, and phenotypic traits. In contrast, causal integration strategies distinguish predictive associations from biologically plausible regulatory mechanisms (Ding et al., 2025; Fu et al., 2022; Haas et al., 2023). The choice of integration strategy should reflect study objectives, sample size, data quality, computational capacity, biological complexity, and the desired balance between predictive performance and interpretability. Hybrid analytical frameworks that combine complementary approaches offer the greatest flexibility for translating multidimensional biological data into robust, interpretable predictive models (Ding et al., 2025; Fu et al., 2022) (Table 2). Integration of genomic, transcriptomic, proteomic, metabolomic, microbiome, physiological, environmental, and production datasets presents substantial computational, statistical, and data-management challenges (An et al., 2025; Cui et al., 2024; Essien et al., 2026; Fu et al., 2022). Although multi-omics approaches provide a more comprehensive representation of complex traits and may improve predictive performance relative to single-data-source models, these advantages are frequently accompanied by increased dimensionality, analytical complexity, computational requirements, infrastructure demands, and cost (An et al., 2025; Cui et al., 2024; Goossens et al., 2022) (Fig. 6). Consequently, future prediction frameworks should optimize computational scalability, algorithmic efficiency, robustness, reproducibility, biological interpretability, and practical implementation alongside accuracy.

**Table 2:** Comparative Evaluation of Predictive Analytical Strategies for Poultry Meat and Egg Quality.

Predictive Strategy	Core Analytical Principle	Key Quality Traits Predicted	Relative Predictive Performance*	Principal Analytical Strength	References
Conventional Genomic Prediction (GEBV)	Uses genome-wide marker effects to estimate breeding values and genetic merit	Growth, carcass yield, egg production, shell quality, feed efficiency	High for moderate-to-high heritability traits	Stable long-term genetic prediction and routine breeding implementation	Aggrey et al., 2010;; [Bibr bib0016][Bibr bib0046]
Regulatory Multi-Omics Models	Integrate genomic variation with downstream regulatory processes	Muscle development, stress response, reproductive physiology, egg formation	Moderate	Connects genetic architecture with dynamic biological regulation	An et al., 2025; Cui et al., 2024; Gu et al., 2024
Functional Phenotype Prediction Models	Incorporate metabolic and physiological signatures reflecting current physiological state	Oxidative stability, flavor development, metabolic efficiency, nutrient utilization	Moderate–High	Captures functional phenotype expression beyond genetic potential	Beauclercq et al., 2018; Ge et al., 2022; Greene et al., 2020
Host–Microbiome Predictive Systems	Integrate host and microbial information to model adaptive biological interactions	Gut health, nutrient efficiency, resilience, environmental adaptation	Moderate	Represents microbiome-mediated influences on productivity and quality	Bernard et al., 2024; Dai et al., 2022; Haas et al., 2023
AI/ML-Assisted Multi-Omics Systems	Apply machine-learning algorithms to identify nonlinear patterns across high-dimensional datasets	Multi-trait quality prediction, resilience, production stability	High (proof-of-concept settings)	Detects complex predictive relationships beyond conventional statistical models	Chin et al., 2023; Ding et al., 2025; Dhungana et al., 2025
Reduced-Feature Biomarker Models	Use a limited set of highly informative biomarkers for prediction	Meat quality, oxidative status, metabolic performance, resilience	Moderate–High	Cost-effective implementation with simplified analytical requirements	Beauclercq et al., 2017; Cui et al., 2024; Greene et al., 2020
Environment-Integrated Prediction Systems	Combine biological indicators with sensor-derived environmental information in real time	Heat-stress resilience, welfare status, physiological stability, quality consistency	Moderate	Continuous monitoring and adaptive decision support under dynamic production conditions	Coble et al., 2014; Doornweerd et al., 2024; Elwakeel, 2025
Digital-Twin and Hybrid Decision-Support Frameworks	Integrate biological, environmental, and production information into dynamic predictive simulations	Whole-system performance, quality consistency, health, and sustainability outcomes	Emerging	Enables scenario testing, adaptive management, and systems-level optimization	Doornweerd et al., 2024; Elwakeel, 2025; Essien et al., 2026
Federated-Learning and Distributed Prediction Systems	Train predictive models across multiple datasets without centralized data sharing	Multi-population prediction, cross-environment robustness, large-scale benchmarking	Emerging	Improves scalability, privacy preservation, and collaborative model development	Essien et al., 2026

Footnote: Relative predictive performance represents a qualitative synthesis of current evidence and should not be interpreted as a direct quantitative comparison among analytical approaches.

GEBV, genomic estimated breeding value.

**Table 3:** Comparative validated predictive performance metrics across genomic, multi-omics, and AI approaches for poultry meat and egg quality traits.

Study	Application Category	Trait Predicted	Data Type	Model	Metric Type	Reported Performance	Evidence Tier
Wolc et al., 2011	Egg production	Egg production traits	Genomics	GBLUP	Predictive correlation	0.32–0.65	Commercial validation
Heidaritabar et al., 2015	Egg production and quality	Egg production and quality traits	Genomics (SNP/WGS)	Bayesian Models / GBLUP	Predictive accuracy	0.30–0.60	Multi-population validation
Heidaritabar et al., 2016	Egg production and quality	Egg production and quality traits	Genomics	GBLUP / Genomic Selection	Predictive accuracy	0.35–0.60	Multi-population validation
Calus et al., 2014	Egg production	Layer performance traits	Genomics	Multi-line GBLUP	Predictive accuracy	0.40–0.76	Multi-population validation
Weng et al., 2016	Production performance	Production and efficiency traits	Genomics	GBLUP / BayesB	Predictive correlation	0.20–0.60	Independent validation
Yan et al., 2018	Egg production	Egg production traits	Genomics	single-step genomic best linear unbiased prediction (ssGBLUP)	Predictive ability	0.30–0.55	Independent validation
Bermann et al., 2021	Feed efficiency	Growth and feed-efficiency traits	Genomics	SsGBLUP	Predictive accuracy	0.30–0.55	Independent validation
Beauclercq et al., 2018	Digestive efficiency	AMEn and digestive efficiency	Metabolomics (NMR/LC-MS)	PLS Regression	R² / Q²	0.50–0.60	Experimental validation
Diaz-Sanchez et al., 2019	Feed-efficiency classification	Feed-efficiency classification	Microbiome (16S rRNA)	Random Forest	ROC-AUC	0.67–0.85	Proof-of-concept
Xu et al., 2021	Disease prediction	Campylobacter prevalence	Farm and environmental data	Random Forest	AUC	0.864–0.902	Independent validation
Zhang et al., 2023	Resilience and growth	Growth and gut resilience	Integrated Multi-Omics	Machine-Learning Integration	Classification accuracy	95.1%	Proof-of-concept
Li et al., 2024	Production performance	Production and efficiency traits	Multi-Omics + ML	Hybrid ML / Genomic Models	R² / Correlation	0.35–0.75	Proof-of-concept
Serva et al., 2023	Meat quality	Meat-quality traits	NIR Phenomics	ML + Chemometrics	Cross-validated R² (R²cv)	0.98–0.99	Cross-validation
Jia et al., 2026	Egg production	Longitudinal egg-production traits	Genomics + Phenotypes	ssGBLUP + Random Regression	Relative accuracy improvement	+2.9–6.8%	Multi-population validation

Footnote: Reported performance metrics were extracted from original studies and are not directly comparable across traits, populations, datasets, validation strategies, or analytical frameworks. SNP, single nucleotide polymorphism; WGS, whole-genome sequencing; GBLUP, genomic best linear unbiased prediction; AMEn, nitrogen-corrected apparent metabolizable energy; NMR, nuclear magnetic resonance; LC-MS = liquid chromatography–mass spectrometry; PLS, partial least-squares; ROC-AUC, receiver operating characteristic area under the curve; AUC, area under the curve; R², coefficient of determination; Q², predictive coefficient of determination.

**Table 4:** Precision nutrition and adaptive intervention strategies for optimization of poultry meat and egg quality.

Intervention Strategy	Primary Target Phenotype	Biological Basis	Principal Quality Outcome	Translational Readiness	Reference
Precision amino acid balancing and genotype-guided protein optimization	Growth efficiency, muscle yield, meat quality, nitrogen utilization	Nutrigenomics, amino-acid signaling, protein accretion, mitochondrial metabolism, and nutrient partitioning	Improved muscle accretion, feed efficiency, meat quality, and nitrogen-use efficiency	High	de Freitas Dionizio et al., 2021; Kidd et al., 2021
Functional lipid supplementation and omega-3 enrichment	Fatty-acid composition, oxidative stability, yolk quality, nutritional value	Lipid metabolism, membrane remodeling, inflammatory regulation, and fatty-acid signaling	Improved fatty-acid profile, oxidative stability, yolk quality, and nutritional value	Moderate–High	Ehr et al., 2017; Elkin et al., 2015
Microbiome-targeted nutritional modulation (probiotics, prebiotics, synbiotics, phytogenics)	Gut health, nutrient utilization, immune resilience, environmental adaptation	Host–microbiome interactions, microbial metabolites, intestinal barrier regulation, and immune modulation	Enhanced intestinal integrity, nutrient utilization, immune competence, and production stability	High	Ferrocino et al., 2023; Gao et al., 2017
Precision antioxidant and mitochondrial-support interventions	Oxidative stability, heat tolerance, meat-quality preservation, stress resilience	Redox regulation, mitochondrial bioenergetics, oxidative-stress mitigation, and antioxidant defense systems	Reduced oxidative damage, improved meat stability, and enhanced physiological resilience	Moderate–High	Greene et al., 2025; Ouyang et al., 2022
Mineral and vitamin optimization for egg-quality enhancement	Shell strength, shell quality, albumen stability, reproductive performance	Calcium transport, mineral metabolism, eggshell biomineralization, and reproductive physiology	Improved eggshell integrity, albumen quality, laying consistency, and reproductive performance	High	Feng et al., 2023; Garcia-Mejia et al., 2024
Environmentally adaptive precision feeding and stress-responsive nutritional programming	Heat-stress resilience, metabolic stability, productivity under environmental challenge	Endocrine adaptation, stress physiology, microbiome dynamics, and metabolic regulation	Improved thermotolerance, physiological stability, productivity, and product quality under stress	Moderate	Emami et al., 2021; Gao et al., 2025c
Multi-component precision intervention programs integrating nutrition, microbiome modulation, and environmental management	Meat quality, egg quality, welfare, resilience, production efficiency	Systems biology, integrated physiology, and host–microbiome–environment interactions	Simultaneous optimization of quality, welfare, resilience, and production traits	Emerging–Moderate	Elbaz et al., 2025; He et al., 2023

Footnote: Translational readiness reflects the current level of scientific validation and commercial implementation within poultry production systems. Omega-3, omega-3 polyunsaturated fatty acids.

**Table 5:** Environmental stressors, multi-omics signatures, and resilience biomarkers associated with poultry meat and egg quality.

Environmental Stressor	Primary Biological Response	Representative Multi-Omics Signatures	Representative Resilience Biomarkers	Product-Quality Consequences	Reference
Chronic heat stress and thermal fluctuation	Impaired thermoregulation, oxidative stress, mitochondrial dysfunction, reduced feed intake	HSP70/HSP90 induction, oxidative-metabolite imbalance, mitochondrial proteomic disruption, microbiome restructuring	Heat-shock proteins, superoxide dismutase (**SOD**) activity, glutathione status, mitochondrial-function indicators	Reduced meat oxidative stability, increased lipid oxidation, impaired eggshell quality, decreased albumen quality	An et al., 2025; Deng et al., 2022; Emami et al., 2021
Selection-associated metabolic and oxidative stress	Metabolic overload, reactive oxygen species accumulation, impaired bioenergetics	Oxidative-stress transcriptomic signatures, mitochondrial dysfunction markers, lipidomic remodeling	MDA, antioxidant-enzyme activity, mitochondrial integrity markers	Wooden breast, white striping, reduced meat quality, impaired processing performance	Abasht et al., 2016; Greene et al., 2020; Petracci et al., 2015
Intestinal dysbiosis and microbial instability	Reduced microbial diversity, impaired nutrient utilization, intestinal inflammation	Microbial community disruption, SCFA depletion, immune–microbiome signaling alterations	Microbial diversity indices, butyrate concentration, intestinal tight-junction markers	Reduced feed efficiency, inconsistent nutrient utilization, variable product quality	Dai et al., 2022; Gao et al., 2017; Oakley et al., 2014
Nutritional and metabolic imbalance	Amino-acid dysregulation, lipid imbalance, endocrine disruption, metabolic inefficiency	Nutrigenomic alterations, lipidomic remodeling, metabolomic pathway perturbations	Plasma amino-acid profiles, lipid-metabolism biomarkers, metabolic-regulation indicators	Reduced muscle integrity, altered egg composition, decreased oxidative stability	Beauclercq et al., 2018; Ge et al., 2022; Kidd et al., 2021
Housing and environmental management stress	Respiratory stress, immune activation, impaired environmental adaptation	Stress-associated metabolomic shifts, inflammatory proteins, environmental microbiome variation	Corticosterone concentration, heterophil-to-lymphocyte ratio, immune-homeostasis indicators	Reduced flock uniformity, shell-quality deterioration, variable production performance	Doornweerd et al., 2024; Kim et al., 2021
Chronic inflammatory stress	Persistent immune activation, cytokine imbalance, oxidative tissue injury	Immune transcriptomics, inflammatory metabolomics, cytokine-associated proteomics	IL-6, TNF-α, antioxidant-defense biomarkers, inflammatory-resolution markers	Reduced oxidative stability, shortened shelf life, altered flavor characteristic	Elgendey et al., 2022; Richards et al., 2020
Climate variability and environmental fluctuation	Endocrine fluctuation, physiological adaptation, metabolic reprogramming	Longitudinal multi-omics variability, adaptive transcriptomics, metabolic-drift signatures	Corticosterone dynamics, physiological-stability indicators, adaptive-response biomarkers	Variable productivity and product quality depending on adaptive capacity	An et al., 2025; Mei et al., 2025

Footnote: Representative biomarkers are illustrative examples derived from current poultry systems-biology literature and should not be interpreted as universally validated predictors across all populations or production environments. HSP70, heat shock protein 70; HSP90, heat shock protein 90; SCFA, short-chain fatty acid; IL-6, interleukin-6; TNF-α, tumor necrosis factor-alpha.

**Table 6:** Economic, infrastructure, regulatory, and implementation barriers to commercial adoption of precision poultry technologies.

Technology	Major Cost Drivers	Infrastructure Requirements	Workforce Expertise Requirements	Scalability Constraints	Regulatory / Data Governance Challenges	Primary Adoption Bottleneck	References
Genomic Selection (GEBV)	Genotyping, phenotyping, reference-population development, genomic evaluation programs	Large-scale breeding databases, genomic information systems, computing infrastructure	Quantitative genetics, breeding, statistical genomics, bioinformatics	Continuous recalibration across populations, environments, and generations	Data ownership, intellectual-property protection, breeding-company confidentiality	High initial investment and long-term maintenance of reference populations	Aggrey et al., 2010; Meuwissen et al., 2001; Wolc et al., 2016
Transcriptomics	RNA sequencing, sample preparation, computational analysis, data storage	Molecular laboratories, sequencing platforms, bioinformatics pipelines	Molecular biology, functional genomics, computational biology	Limited routine commercial deployment due to cost and analytical complexity	Lack of standardized protocols, pipelines, and cross-platform reproducibility	High analytical cost and operational complexity	Abasht et al., 2016; Beauclercq et al., 2017
Proteomics	Mass spectrometry instrumentation, consumables, sample processing	Advanced proteomics laboratories and analytical platforms	Proteomics, analytical chemistry, computational biology	Limited throughput and inter-laboratory reproducibility	Cross-platform harmonization and validation challenges	Technical complexity and high operational cost	Berri et al., 2019; Dai et al., 2025
Metabolomics	Instrumentation, metabolite identification, spectral database development	Metabolomics platforms, analytical laboratories, computational resources	Metabolomics, analytical chemistry, bioinformatics	Sample-throughput limitations and high analytical costs	Standardization of metabolite annotation and analytical workflow	Limited cost-effective routine deployment	Beauclercq et al., 2018; Ge et al., 2022
Microbiome Profiling	Sequencing, longitudinal sampling, bioinformatics processing	Sequencing facilities, microbiome databases, computational infrastructure	Microbial ecology, microbiomics, bioinformatics	Population- and environment-specific microbial responses reduce transferability	Standardization, interoperability, and reproducibility limitation	Variable external validity across production systems	Aruwa et al., 2021; Bernard et al., 2024; Dai et al., 2022
Precision Nutrition Platforms	Sensors, feed-formulation technologies, monitoring systems, decision-support software	Integrated digital farm-management systems and production databases	Poultry nutrition, precision management, data interpretation	Biological and management variability among farms	Limited standardization of implementation frameworks	Demonstration of consistent economic return under commercial conditions	Cao et al., 2024; Abd El-Hack et al., 2025
Environmental Phenomics and Sensor Networks	Sensor acquisition, installation, calibration, maintenance, connectivity	Real-time monitoring systems, cloud infrastructure, communication networks	Sensor engineering, environmental monitoring, data analytics	Large-scale deployment and maintenance requirements	Data ownership, privacy, storage, and long-term management concerns	Hardware deployment and maintenance costs	Doornweerd et al., 2024; Elwakeel, 2025
Biosensor-Based Monitoring	Biosensor development, validation, calibration, replacement	Continuous monitoring infrastructure and diagnostic integration systems	Sensor operation, maintenance, analytical interpretation	Long-term durability, reliability, and calibration consistency	Validation, certification, and quality-control requirements	Reliability and scalability under commercial conditions	Balthazar et al., 2025; Elwakeel, 2025
Multi-Omics Integration Frameworks	Multi-platform data generation, storage, integration, and computing	High-performance computing, interoperable databases, data repositories	Systems biology, bioinformatics, statistics, AI	Integration of heterogeneous high-dimensional datasets	Data sharing, interoperability, governance, and reproducibility limitation	Computational burden and lack of analytical standardization	Argelaguet et al., 2018, 2020; Fu et al., 2022
Machine-Learning Decision Systems	Model development, training, validation, updating, computational resources	Digital data ecosystems, cloud computing, decision-support platforms	AI, ML, statistics, poultry domain expertise	Limited generalizability across breeds, environments, and management systems	Transparency, accountability, validation, and governance requirements	Limited external validation under commercial production conditions	Ayoola et al., 2023; Benefo et al., 2024; Chen et al., 2025
XAI	Development and validation of interpretable predictive frameworks	Advanced computational and analytical infrastructure	AI, causal inference, systems biology, model interpretation	Balancing interpretability with predictive accuracy and robustness	Emerging transparency, governance, and accountability requirements	Limited poultry-specific validation evidence	Ballard et al., 2024; Chen et al., 2025
Reduced-Feature Biomarker Panels	Biomarker discovery, assay optimization, validation, deployment	Targeted diagnostic platforms and laboratory infrastructure	Laboratory diagnostics, analytical sciences, data interpretation	Biomarker robustness across populations and environments	Assay validation, standardization, and regulatory acceptance	Independent multi-population validation	Beauclercq et al., 2017; Berri et al., 2019
Digital-Twin Production Systems	Model construction, data integration, maintenance, continuous updating	Continuous biological, environmental, and production-monitoring infrastructure	Systems modeling, AI, production analytics, computational science	Limited availability of validated biological models	Data governance, interoperability, transparency, and accountability	Lack of validated commercial-scale implementations	Elwakeel, 2025; Essien et al., 2026
Integrated Decision-Support Systems	Software integration, maintenance, user training, operational support	Farm-wide digital infrastructure integrating biological, environmental, and production data	Multidisciplinary expertise spanning biology, analytics, engineering, and management	Compatibility across diverse production systems	Data governance, interoperability, cybersecurity, and data-sharing requirements	Demonstration of measurable economic benefit under commercial conditions	Elwakeel, 2025; Essien et al., 2026

Footnote: Adoption barriers are context dependent and may vary according to production scale, technological maturity, regulatory requirements, infrastructure availability, and economic conditions. GEBV, genomic estimated breeding value; FAIR, Findable, Accessible, Interoperable, and Reusable.

**Fig. 7:**
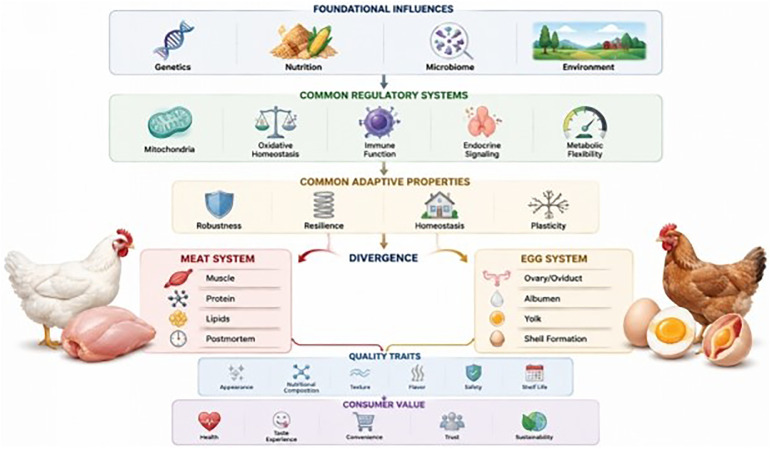
**Shared and divergent biological architectures of poultry product quality:** Poultry meat and egg quality originate from distinct tissue-specific biological processes but are regulated by a common systems biology foundation. Genetic, nutritional, microbial, environmental, and physiological factors influence shared regulatory systems, including mitochondrial function, oxidative homeostasis, immune competence, endocrine signaling, and metabolic flexibility, which collectively govern adaptive system properties such as robustness, resilience, and homeostatic stability. From this common adaptive architecture, biological regulation diverges into muscle-specific pathways governing meat-quality traits (tenderness, water-holding capacity, color, and flavor) and reproductive pathways governing egg-quality traits (shell strength, albumen quality, yolk characteristics, and nutrient composition). Despite their distinct phenotypic manifestations, both product categories emerge from a shared, integrated adaptive biological system architecture, providing a systems biology rationale for evaluating poultry product quality within a unified framework.

AI and ML methods are increasingly applied to genomic prediction, biomarker discovery, disease surveillance, precision nutrition, automated phenotyping, welfare assessment, environmental monitoring, and integrated decision support (Ayoola et al., 2023; Bai et al., 2023; Dhungana et al., 2025; Doornweerd et al., 2024; Essien et al., 2026). By integrating large-scale genomic, molecular, phenotypic, environmental, and management datasets, these approaches have substantial potential to improve prediction of production, health, welfare, and meat- and egg-quality traits while enabling more responsive, data-driven management (Ayoola et al., 2023; Cui et al., 2024; Doornweerd et al., 2024; Essien et al., 2026).

Nevertheless, successful implementation requires models that are not only accurate but also biologically interpretable, computationally efficient, reproducible, externally validated, and robust across diverse genetic populations, production environments, and management systems (Alves et al., 2024; Chu et al., 2019; Cui et al., 2024; Goossens et al., 2022). Major barriers to widespread adoption remain limited external validation, reduced transferability, integration of heterogeneous datasets, and the limited interpretability of complex machine-learning models.

The emergence of XAI, biologically informed ML algorithms, causal inference, graph-based learning, and mechanistic network integration provides promising opportunities to improve model transparency, identify biologically meaningful predictors, and strengthen confidence in AI-assisted decision-making. These complementary approaches combine predictive performance with mechanistic interpretability, which can accelerate the development of reliable, transparent, and commercially deployable systems for precision breeding, nutrition, health management, environmental control, and production optimization. However, continued progress depends on rigorous external validation, standardized benchmarking, transparent reporting, and multidisciplinary collaboration. These measures ensure that AI-enabled prediction systems remain biologically credible, operationally practical, and economically sustainable.

### Scalable precision poultry systems

Long-term adoption of precision poultry technologies depends on effective integration of biological, environmental, management, and production information within economically viable decision-support frameworks capable of operating across diverse commercial settings (Chu et al., 2019; Doornweerd et al., 2024; Elwakeel, 2025; Essien et al., 2026). Advances in AI, precision phenotyping, biosensors, automated monitoring, and multimodal data integration increasingly enable real-time assessment of poultry health, welfare, performance, and product quality (Balthazar et al., 2025; Dhungana et al., 2025; Doornweerd et al., 2024; Elwakeel, 2025; Essien et al., 2026). Together, these technologies support adaptive management, continuous optimization, and evidence-based decision-making, improving production efficiency, resource utilization, and system resilience.

Despite these advances, widespread implementation remains constrained by interoperability, infrastructure requirements, data standardization, economic feasibility, workforce expertise, cybersecurity, and limited validation across diverse production environments. Accordingly, rigorous multi-environment field validation, independent performance assessment, standardized data architectures, and demonstration of economic value remain essential prerequisites for large-scale deployment (Doornweerd et al., 2024; Elwakeel, 2025; Essien et al., 2026; Goossens et al., 2022).

**Fig. 8:**
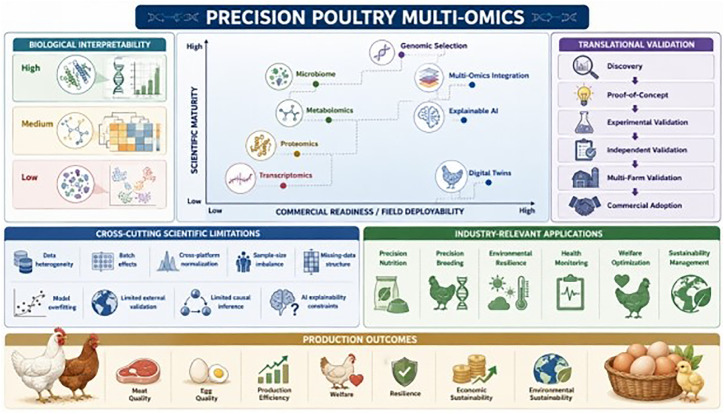
**Translational evidence landscape for precision poultry multi-omics:** Multi-omics–enabled precision poultry technologies differ markedly across dimensions of scientific maturity, biological interpretability, validation status, and commercial readiness. The framework illustrates the progression from discovery-oriented omics research toward validated field applications and commercial implementation. Technologies must advance through successive stages of experimental, independent, and multi-farm validation prior to routine industry adoption. Cross-cutting challenges, including data heterogeneity, batch effects, cross-platform normalization, overfitting, limited causal inference, restricted external validation, and AI interpretability, remain major barriers to translation. Successful implementation of these technologies supports precision nutrition, precision breeding, environmental resilience, health monitoring, welfare optimization, and sustainability management, ultimately contributing to improvements in poultry meat quality, egg quality, production efficiency, animal welfare, economic viability, and environmental sustainability.

Future progress will require scalable precision-poultry platforms that combine predictive accuracy, biological interpretability, operational feasibility, interoperability, and seamless integration into routine production workflows (Fig. 8). Such platforms will be fundamental for translating multi-omics discoveries, precision-production technologies, and AI-enabled decision support into reliable, cost-effective, and commercially deployable solutions that enhance productivity, animal welfare, product quality, and the long-term sustainability of next-generation poultry production.

## KNOWLEDGE GAPS AND RESEARCH LIMITATIONS

Despite significant advances in poultry systems biology, several critical biological, methodological, and translational challenges continue to hinder the development of robust, mechanistically interpretable, and broadly generalizable predictive frameworks. These arise from the complexity of poultry biological systems, the heterogeneity of multi-omics studies, difficulties in integrating high-dimensional datasets, limited capacity for causal inference, insufficient external validation, variability in analytical reproducibility, and uncertainty regarding the temporal and environmental stability of predictive biomarkers and models (Bernard et al., 2024; Cui et al., 2024; Essien et al., 2026; Fu et al., 2022; Goossens et al., 2022).

Many reported molecular associations also remain population-, breed-, or context-specific. This restricts transferability across genetic backgrounds, production systems, and commercial environments because of genotype-by-environment (G × E) interactions, environmental heterogeneity, and inherent biological variation (Bernard et al., 2024; Chu et al., 2019; de Kinderen et al., 2023).

Addressing these limitations will require harmonized phenotyping protocols, standardized analytical workflows, longitudinal experimental designs, multi-environment validation studies, and mechanistically informed modeling approaches. Together, these priorities provide the foundation for translating multi-omics discoveries into reliable, biologically interpretable, and scalable decision-support systems for next-generation precision poultry production (Table 9).

### Biological Complexity and Emergence

The phenotypes of poultry emerge through the dynamic interaction of genetic architecture, physiological regulation, microbiome composition, nutrition, immune function, management practices, and environmental conditions. These interconnected processes operate across multiple biological scales, collectively determining growth, health, resilience, feed efficiency, and the quality of meat and eggs (Abasht et al., 2016; Dai et al., 2022; Gao et al., 2024; Gautam et al., 2024; Gu et al., 2024).

These biological relationships are inherently context-dependent. They are substantially influenced by genotype-by-environment (G × E) interactions, developmental stage, nutritional status, microbiome composition, and production conditions (Chu et al., 2019; de Kinderen et al., 2023; Gao et al., 2025b; Gautam et al., 2024). Consequently, biomarkers identified within a particular experimental setting may not consistently retain predictive value across breeds, production environments, or management systems.

This context dependence complicates biomarker discovery, biological interpretation, and model transferability. Increasing evidence indicates that robust prediction requires integration of molecular, physiological, microbial, and environmental interactions rather than reliance on isolated biomarkers (Cui et al., 2024; Ding et al., 2025; Fu et al., 2022; Ghafouri et al., 2021).

Progress in this area will rely on longitudinal investigations, multi-environment validation, and systems-level analytical approaches capable of distinguishing stable biological determinants from transient or context-specific responses. Such efforts are essential for developing predictive frameworks that remain biologically meaningful, robust, and generalizable across diverse poultry populations and production environments.

### Data integration and methodological diversity

One of the principal challenges in poultry systems biology remains the integration of genomic, transcriptomic, proteomic, metabolomic, microbiome, phenotypic, and environmental information. Differences in data structure, dimensionality, measurement scales, analytical platforms, and quality complicate systems-level interpretation while limiting interoperability, reproducibility, and cross-study integration (Ding et al., 2025; Fu et al., 2022; Goossens et al., 2022).

Methodological diversity further arises from differences in experimental design, population characteristics, sample collection, biospecimen processing, sequencing technologies, normalization procedures, and bioinformatics workflows. These variations introduce batch effects, technical bias, and analytical variability that reduce reproducibility, limit comparability, and constrain large-scale data harmonization (Ding et al., 2025; Fu et al., 2022; Goossens et al., 2022).

Another important limitation is the imbalance between the large number of measured molecular variables and the comparatively modest sample sizes available in many poultry investigations. High-dimensional datasets increase susceptibility to overfitting, unstable feature selection, model overparameterization, and inflated estimates of predictive performance, particularly when independent external validation is unavailable (Alves et al., 2024; Ding et al., 2025; Wolc et al., 2011).

Future advances require harmonized phenotyping protocols, standardized analytical workflows, interoperable data infrastructures, larger multi-population datasets, rigorous external validation, and transparent reporting standards. Continued refinement of integrative analytical frameworks that can combine molecular, physiological, microbiome, environmental, and production information will improve biological interpretation, reproducibility, predictive accuracy, and usefulness for translation (Table 7).

**Table 7:** Methodological limitations and best-practice solutions for predictive multi-omics integration in poultry meat and egg quality research.

Methodological Challenge	Scientific Consequence	Best-Practice Mitigation Strategy	Impact on Predictive Reliability	References
High-dimensional feature space (p ≫ n)	Overfitting, unstable feature selection, inflated prediction accuracy, poor biological generalization	Regularization approaches, dimensionality reduction, biologically informed feature selection, larger multi-line reference populations	Improves model robustness, stability, and generalizability	Alves et al., 2024; Daetwyler et al., 2013
Batch effects and platform heterogeneity	Technical variation obscures biological signals and reduces reproducibility	Standardized sample processing, quality-control pipelines, harmonized analytical workflows, cross-platform calibration	Improves reproducibility and biomarker reliability	Conesa and Beck, 2019; Fu et al., 2022
Inconsistent normalization across omics layers	Distorted biological interpretation and biased integrated models	Unified preprocessing pipelines, cross-platform normalization, sensitivity analyses, reference-based scaling	Enhances integration accuracy and analytical consistency	Argelaguet et al., 2018, 2020
Fragmented and non-interoperable data architectures	Reduced ability to integrate genomic, transcriptomic, metabolomic, microbiome, and phenotypic datasets	FAIR principles, ontology harmonization, centralized repositories, interoperable databases, cloud-based infrastructures	Facilitates systems-level integration and reproducibility	Fu et al., 2022; Goossens et al., 2022
Limited interpretability of AI and machine-learning models	Reduced biological explainability and mechanistic understanding	Explainable AI, biologically constrained ML, hybrid mechanistic-AI frameworks, causal modeling	Improves transparency, interpretability, and user confidence	Ayoola et al., 2023; Ballard et al., 2024
Insufficient external validation across populations and environments	Poor transferability and inconsistent performance across production systems	Independent validation cohorts, geographically diverse populations, prospective benchmarking studies	Strengthens deployment readiness and predictive robustness	Chu et al., 2019; de Kinderen et al., 2023
Temporal instability of biomarkers	Prediction accuracy changes across age, physiological state, and environmental conditions	Longitudinal sampling, repeated validation, dynamic recalibration, time-series analyses	Improves long-term predictive stability	Bendikov-Bar et al., 2021; Gao et al., 2025b
Scalability constraints for real-time multi-omics implementation	High computational burden and limited commercial deployment	Reduced biomarker panels, digital phenotyping, sensor integration, edge computing, multimodal monitoring platforms	Improves operational feasibility and industrial adoption	Berri et al., 2019; Doornweerd et al., 2024; Essien et al., 2026
Limited biological standardization of phenotypes	Inconsistent trait definitions and reduced comparability among studies	Harmonized phenotyping protocols, validated measurements, standardized ontologies, shared reference populations	Improves benchmarking and reproducibility	Berger et al., 2022; Le Bihan-Duval et al., 2008; Mignon-Grasteau et al., 2015
Predominance of correlative rather than causal inference frameworks	Associations identified without confirmation of biological mechanisms	Functional validation, intervention studies, perturbation experiments, gene-editing approaches, causal-network analyses	Strengthens mechanistic confidence and biomarker utility	Beauclercq et al., 2017, 2018; Ding et al., 2026; Haas et al., 2023

Footnote: Methodological limitations frequently interact and collectively influence predictive accuracy, biological interpretation, reproducibility, and translational applicability. p ≫ n, number of variables greatly exceeds number of samples; FAIR, Findable, Accessible, Interoperable, and Reusable.

**Table 8:** Translational roadmap for multi-omics–enabled poultry meat and egg quality prediction.

Translational Phase	Scientific Objective	Critical Requirement	Validation Milestone	Advancement Criterion	References
Biomarker Discovery	Identify genomic, transcriptomic, proteomic, metabolomic, lipidomic, and microbiome signatures associated with meat and egg quality traits	Standardized phenotyping and well-characterized experimental populations	Reproducible molecular associations across independent datasets	Robust candidate biomarker identification	Abasht et al., 2016; Beauclercq et al., 2017; Cui et al., 2024
Biological Validation	Establish mechanistic relevance and causal relationships between candidate biomarkers and quality phenotypes	Functional validation and independent replication studies	Consistent biological effects across genetic backgrounds and production environments	Mechanistically supported biomarkers	Beauclercq et al., 2018; Ding et al., 2026; Haas et al., 2023
Multi-Omics Integration	Integrate molecular, physiological, microbiome, and environmental information into unified predictive frameworks	Harmonized datasets and standardized analytical pipelines	Stable cross-platform integration performance	Robust systems-level predictive models	Ding et al., 2025; Fu et al., 2022; Ghafouri et al., 2021
Predictive Model Development	Develop accurate and biologically interpretable prediction systems for quality and resilience traits	Large-scale multi-omics and phenotypic datasets	Robust internal and external validation	High predictive accuracy with biological interpretability	Alves et al., 2024; Ballard et al., 2024; Wolc et al., 2011
Cross-System Validation	Evaluate model generalizability across breeds, environments, production systems, and temporal scales	Multi-location and multi-population validation cohorts	Consistent performance under diverse commercial conditions	Reliable cross-environment prediction	Chu et al., 2019; de Kinderen et al., 2023
Biomarker Simplification	Reduce complex molecular signatures into cost-effective and deployable biomarker panels	Feature-selection and model-reduction strategies	Retention of predictive performance after dimensional reduction	Compact, scalable, and robust biomarker panels	Berger et al., 2022; Berri et al., 2019
Operational Implementation	Integrate validated predictive tools into breeding, nutrition, health, and management workflows	Interoperable data systems and interpretable analytical platforms	Successful incorporation into routine decision-support workflows	Practical and reproducible field utilization	Bortoluzzi et al., 2025; Fu et al., 2022; Goossens et al., 2022
Adaptive Precision Production	Enable continuous optimization through integration of omics, sensors, AI, and real-time monitoring technologies	Dynamic data acquisition and continual-learning architectures	Real-time predictive adaptation and quality optimization	Real-time adaptive precision production systems	Bhujel et al., 2024; Doornweerd et al., 2024; Elwakeel, 2025; Essien et al., 2026

Footnote: The translational roadmap outlines a conceptual progression from biomarker discovery to adaptive precision-production systems and requires iterative validation at each developmental stage.

**Table 9:** Hierarchy of Evidence Supporting Precision-Poultry Prediction of Meat and Egg Quality.

Evidence Category	Typical Study Design	Strength of Inference	Reproducibility and External Validity Potential	Translational Value	Evidence Level	References
Independent multi-population validation studies	Validation across breeds, environments, and production systems	Very High	Very High	Very High	I	Chu et al., 2019; de Kinderen et al., 2023; Wolc et al., 2011
Commercial deployment studies	Industry-scale implementation and performance evaluation	Very High	High	Very High	I	Bortoluzzi et al., 2025; Goossens et al., 2022
Prospective longitudinal cohort studies	Repeated measurements across production cycles	High	High	High	II	Bendikov-Bar et al., 2021; Essien et al., 2026
Multi-environment experimental studies	Controlled validation under diverse environmental conditions	High	High	High	II	Chu et al., 2019; de Kinderen et al., 2023
Controlled intervention studies	Nutritional, microbiome, management, or environmental manipulation experiments	Moderate–High	Moderate–High	High	III	Beauclercq et al., 2018; Deng et al., 2022; Gao et al., 2025c
Multi-omics studies with independent external validation	Integrated genomic, transcriptomic, proteomic, metabolomic, and microbiome datasets with independent testing	Moderate–High	Moderate	Moderate–High	III	Cui et al., 2024; Ding et al., 2025; Gao et al., 2025b
Single-population omics studies	Genomic, transcriptomic, proteomic, metabolomic, lipidomic, or microbiome analyses within one population	Moderate	Moderate	Moderate	IV	Dermane et al., 2024; Ge et al., 2022; Greene et al., 2020
Biomarker discovery studies	Identification of candidate predictive markers without independent validation	Low–Moderate	Low–Moderate	Moderate	V	Abasht et al., 2016; Beauclercq et al., 2017
Cross-sectional association studies	Correlative analyses without temporal or experimental validation	Low	Low	Low	VI	Gao et al., 2024; Gautam et al., 2024
In silico predictive studies without experimental validation	Algorithm development and computational modeling only	Very Low	Unknown	Low	VII	Ballard et al., 2024
Conceptual frameworks and expert opinion	Perspectives, reviews, methodological frameworks, and theoretical models	Very Low	Not established	Low	VIII	Conesa and Beck, 2019; Goossens et al., 2022

Footnote: Evidence levels were adapted from translational-research principles and reflect decreasing inferential strength, reproducibility, external validity, and translational confidence from Level I to Level VIII.

### Causality and mechanistic understanding

Many poultry multi-omics investigations primarily identify statistical associations and candidate biomarkers rather than experimentally validated causal mechanisms, which is a fundamental limitation. Genomic, transcriptomic, proteomic, metabolomic, and microbiome analyses reveal molecular signatures associated with meat quality, egg quality, feed efficiency, health, resilience, and other economically important traits. However, association alone does not establish biological causation (Beauclercq et al., 2018; Cui et al., 2024; Ding et al., 2025).

Likewise, high predictive accuracy should not be interpreted as evidence of mechanistic understanding. Molecular features associated with a phenotype may represent causal drivers, downstream responses, compensatory adaptations, or correlated biomarkers embedded within complex regulatory networks. Distinguishing among these possibilities requires additional experimental and analytical evidence (Beauclercq et al., 2018; Ding et al., 2026; Haas et al., 2023).

Strengthening causal inference will require the integration of multi-omics analyses with functional validation, controlled intervention studies, longitudinal experiments, perturbation analyses, and emerging causal-network modeling approaches. Together, these complementary strategies can identify biological pathways linking genomic variation, molecular regulation, physiological adaptation, microbiome dynamics, and environmental responses with economically important production traits.

Although considerable methodological progress has been achieved, a substantial gap remains between association-based prediction and experimentally validated causation. Closing this gap is essential for developing biologically interpretable, robust, generalizable, and commercially deployable predictive systems for precision poultry production (Table 10).

**Table 10:** Major scientific controversies and unresolved questions in precision poultry systems biology.

Topic	Evidence Supporting Position A	Evidence Supporting Position B	Current State of Evidence	Key Unresolved Question	References
High-dimensional multi-omics integration vs reduced biomarker panels	Integrated multi-omics captures complementary genomic, transcriptomic, metabolomic, microbiome, and physiological information underlying complex quality traits	Reduced biomarker panels may retain much of the predictive signal while improving scalability, cost-efficiency, and interpretability	Evidence remains trait- and application-dependent; no universal superiority has been established	Under which biological and commercial conditions does additional omics complexity generate meaningful predictive gains?	Berri et al., 2019; Ding et al., 2025; Ghafouri et al., 2021
Universal vs context-dependent biomarkers	Some genomic and physiological markers demonstrate predictive value across populations	Biomarker effects frequently vary because of G × E interactions, management practices, age, and production system differences	Strong evidence supports substantial biological context dependence	Can universally transferable biomarkers be identified without sacrificing prediction accuracy?	Chu et al., 2019; de Kinderen et al., 2023; Gao et al., 2025b
Microbiome causality versus association	Gut microbiota consistently associates with growth, metabolism, immunity, nutrient utilization, and product quality traits	Most microbiome studies remain observational and cannot conclusively establish causality	Current evidence remains predominantly correlative despite increasing mechanistic insight	Which microbial taxa, pathways, or metabolites directly drive meat and egg quality variation?	Dai et al., 2022; Gao et al., 2024; Gautam et al., 2024; Haas et al., 2023
Deep-learning prediction versus XAI	Advanced machine-learning methods frequently improve prediction of complex traits in high-dimensional biological datasets	Limited interpretability may reduce biological understanding, validation confidence, and adoption by breeding and production programs	The accuracy–interpretability trade-off remains unresolved	How can predictive performance be maximized without sacrificing biological transparency?	Ballard et al., 2024; Dhungana et al., 2025; Essien et al., 2026
Digital-twin poultry production systems	Precision sensors, computer vision, automated monitoring, and multimodal data streams increasingly enable real-time biological surveillance	Fully integrated digital-twin systems with robust commercial validation remain scarce	Core enabling technologies exist, but comprehensive validation remains limited	What level of biological, environmental, and management integration is necessary to achieve reliable digital twins?	Bhujel et al., 2024; Doornweerd et al., 2024; Elwakeel, 2025; Essien et al., 2026;
Prediction versus biological understanding	High predictive accuracy can often be achieved using ML, genomic prediction, and systems-biology approaches	Mechanistic interpretation and causal inference frequently lag behind predictive performance	A persistent gap remains between prediction and mechanistic understanding	Can predictive systems simultaneously achieve high accuracy, causal inference, and biological interpretability?	Ballard et al., 2024; Ding et al., 2026; Haas et al., 2023; Wolc et al., 2011

Footnote: The controversies summarized represent major scientific frontiers that currently influence the development, interpretation, and implementation of predictive poultry systems biology. G × E, genotype-by-environment interaction.

### Evidence quality and reproducibility

Ensuring reproducibility, comparability, robustness, and external validity remains a central challenge in poultry multi-omics research. Differences in experimental design, population structure, phenotyping methodologies, sampling procedures, analytical platforms, bioinformatics pipelines, statistical analyses, and integration strategies introduce technical and biological variation that complicates cross-study comparison and limits confidence in systems-level interpretation (Ding et al., 2025; Fu et al., 2022; Goossens et al., 2022).

Although substantial progress has been made in identifying molecular, microbial, metabolic, and physiological signatures associated with poultry performance and product quality, many investigations remain restricted to relatively small populations, single production environments, or narrowly defined experimental conditions. Therefore, independent validation across breeds, genetic lines, production systems, geographic regions, and commercial environments remains insufficient for many proposed biomarkers and prediction models (Alves et al., 2024; Chu et al., 2019; de Kinderen et al., 2023).

Additional limitations arise from inconsistent phenotypic definitions, variable measurement protocols, and the absence of universally accepted standards for multi-omics data generation, processing, analysis, and reporting. These deficiencies hinder reproducibility, reduce confidence in biological generalizability, and constrain large-scale integration across independent investigations (Berger et al., 2022; Ding et al., 2025; Fu et al., 2022).

Addressing these challenges requires harmonized phenotyping, standardized analytical workflows, implementation of FAIR data principles, longitudinal study designs, multi-environment validation, transparent reporting, and routine external validation across independent poultry populations. Strengthening these components will substantially improve reproducibility, biological interpretation, predictive robustness, and translational confidence.

### Unresolved scientific controversies

Several important scientific questions remain unanswered. First, it is unclear whether increasing molecular dimensionality consistently improves predictive performance. Although the integration of complementary omics layers can enhance biological resolution, there is limited convincing evidence demonstrating its consistent superiority over parsimonious biomarker panels across diverse poultry populations and commercial environments (Berri et al., 2019; Cui et al., 2024; Ding et al., 2025; Fu et al., 2022). Therefore, determining the optimal balance among biological complexity, prediction accuracy, interpretability, computational efficiency, and implementation cost remains an important research priority.

Second, uncertainty persists regarding the stability and transferability of biomarkers across genetic backgrounds, management systems, production environments, and temporal scales. Multi-population validation studies consistently demonstrate strong G × E interactions that limit universal applicability of many molecular, microbial, metabolic, and physiological signatures (Alves et al., 2024; Chu et al., 2019; de Kinderen et al., 2023).

Thirdly, the relationship between predictive accuracy and mechanistic interpretability remains unclear. While ML approaches often improve the prediction of complex production traits, the underlying biology is frequently difficult to interpret experimentally and apply to breeding, nutrition, or management decisions (Dhungana et al., 2025; Doornweerd et al., 2024; Essien et al., 2026). This challenge has stimulated growing interest in XAI, biologically informed ML, and hybrid mechanistic–data-driven modeling.

A further unresolved question concerns the extent to which multidimensional biological information can be integrated into unified predictive systems. Although systems-level integration has become increasingly feasible, reproducibility, biological generalizability, and independent validation remain substantially greater challenges than data generation itself (Ding et al., 2025; Fu et al., 2022; Goossens et al., 2022).

Overall, current evidence indicates that future advances will depend less on increasing analytical complexity than on improving phenotypic standardization, longitudinal validation, causal inference, mechanistic understanding, multi-environment evaluation, and commercial validation. Ultimately, the success of precision poultry decision-support systems will be determined by reliability, biological interpretability, robustness, scalability, and practical utility rather than molecular dimensionality alone.

### From biomarker discovery to adaptive-system prediction

A growing body of evidence from poultry genomics, complementary omics technologies, precision phenotyping, microbiome research, and systems-level data integration suggests that meat and egg quality arises from the coordinated interaction of genetic architecture, physiological regulation, microbial ecology, nutrition, management, and environmental conditions (Beauclercq et al., 2018; Ding et al., 2025; Fu et al., 2022; Gao et al., 2024; Gautam et al., 2024; Gu et al., 2024). Collectively, these findings demonstrate that no single molecular layer can consistently capture the biological complexity underlying quality-related phenotypes, underscoring the need for integrative, systems-level analytical frameworks.

Consistent experimental evidence implicates mitochondrial function, redox homeostasis, energy metabolism, immune regulation, and host-microbiome interactions as central determinants of variation in meat and egg quality (Beauclercq et al., 2018; Dai et al., 2022; Ding et al., 2026; Gao et al., 2024; Gautam et al., 2024). These findings suggest that biologically relevant biomarkers tend to reflect integrated physiological states and adaptive biological networks rather than isolated molecular signals.

Current evidence supports an adaptive-systems perspective in which poultry quality emerges from coordinated interactions among molecular, cellular, physiological, microbial, and environmental networks operating across multiple biological scales. When analytical approaches capture resilience, adaptive capacity, robustness, and systems-level organization in addition to molecular variation, predictive performance is expected to improve within this framework.

Validation studies further demonstrate that genotype, nutrition, microbiome composition, management, and environmental conditions interact in highly context-dependent ways. These factors contribute to variability in biomarker performance and limit model transferability across poultry populations and production systems (Alves et al., 2024; Chu et al., 2019; de Kinderen et al., 2023; Ding et al., 2025). Although multi-omics integration can improve biological resolution and mechanistic understanding, evidence demonstrating consistent superiority over parsimonious prediction frameworks across diverse commercial environments remains limited.

Conversely, genomic prediction validated across large breeding populations has demonstrated substantial predictive utility for economically important production traits when combined with precision-production systems integrating biologically informed phenotypic and environmental information (Chu et al., 2019; de Kinderen et al., 2023; Doornweerd et al., 2024; Wolc et al., 2011). Emerging evidence further suggests that integrating genomic, microbiome, metabolomic, physiological, and environmental information can improve prediction of complex traits, including feed efficiency, resilience, robustness, and meat and egg quality (Ding et al., 2025; Fu et al., 2022; Haas et al., 2023).

Overall, the field is shifting towards predictive, mechanistically informed, and biologically interpretable systems biology frameworks that move beyond descriptive molecular profiling. Continued progress in this area depends on integrating complementary omics technologies, precision phenotyping, causal inference methodologies, longitudinal validation, and multi-environment experimentation. These approaches will help elucidate the biological mechanisms underlying resilience, adaptation, stability, and production performance. Such advancements will lay the scientific groundwork for robust, scalable, and commercially deployable adaptive decision support systems that can be used in next-generation precision poultry production (Table 11).

**Table 11:** Integrated evidence strength and translational readiness of poultry multi-omics technologies.

Technology	Evidence Base	Independent Validation	Biological Interpretability	Predictive Utility	Commercial Readiness	Principal Limitations	Overall Evidence Grade	References
Genomic selection (GEBV)	Extensive commercial breeding implementation and validation	High	High	High	High	Requires large reference populations and continual recalibration	A	Chu et al., 2019; de Kinderen et al., 2023; Wolc et al., 2011
Integrated genomics–phenomics approaches	Multiple breeding and precision-phenotyping studies	Moderate–High	High	High	Moderate–High	Data harmonization and interoperability challenges	A−	Berger et al., 2022; Doornweerd et al., 2024
Transcriptomics	Extensive poultry mechanistic studies	Moderate	Moderate–High	Moderate	Moderate	Tissue specificity and temporal variability	B+	Beauclercq et al., 2018; Coble et al., 2014; Gu et al., 2024
Proteomics	Strong evidence for characterization of meat and egg quality traits	Moderate	High	Moderate	Moderate	Cross-platform standardization limitations	B+	Berri et al., 2019; Dai et al., 2025
Metabolomics	Strong biomarker discovery and validation evidence	Moderate	High	Moderate–High	Moderate	Environmental sensitivity and biological variability	A−	Beauclercq et al., 2018; Cui et al., 2024; Ge et al., 2022
Lipidomics	Emerging but rapidly expanding evidence base	Low–Moderate	Moderate	Moderate	Moderate	Limited external validation across populations	B	Chin et al., 2023; Guo et al., 2024b
Microbiome profiling	Extensive poultry microbiome and host–microbiome studies	Moderate–High	High	Moderate–High	Moderate–High	Strong population and environmental dependence	A−	Dai et al., 2022; Gao et al., 2024; Gautam et al., 2024
Precision nutrition platforms	Extensive intervention and validation studies	High	High	High	High	Infrastructure and management requirements	A	Gao et al., 2025c; Ghasemi et al., 2025; Guo et al., 2024a
Environmental phenomics	Strong experimental and commercial evidence	High	High	High	High	Sensor installation and maintenance costs	A	Doornweerd et al., 2024; Elwakeel, 2025
Biosensor-based monitoring	Increasing validation and deployment evidence	Moderate–High	High	Moderate–High	Moderate–High	Hardware integration and scalability challenges	A−	Bhujel et al., 2024; Elmessery et al., 2023; Elwakeel, 2025
Multi-omics integration frameworks	Increasing experimental evidence base	Moderate	Moderate	High	Moderate	Computational complexity and harmonization challenges	B+	Ding et al., 2025; Fu et al., 2022; Ghafouri et al., 2021
Machine-learning predictive models	Strong predictive evidence across poultry datasets	Moderate	Moderate	High	Moderate–High	Limited external validation across populations	A−	Ballard et al., 2024; Dhungana et al., 2025; Essien et al., 2026
XAI	Emerging poultry-specific evidence	Low–Moderate	High	Moderate	Moderate	Limited poultry validation and benchmarking studies	B	Ballard et al., 2024; Essien et al., 2026

Footnote: Evidence grades represent qualitative assessments of validation strength, reproducibility, biological plausibility, predictive utility, and commercial readiness based on currently available evidence.

**Table 12:** Proposed adaptive systems trait prediction of poultry quality (ASTPQ) framework.

ASTPQ Component	Candidate Biomarker(s)	Measurement Approach	Expected Biological Outcome	References
Mitochondrial Efficiency	ATP concentration, mitochondrial membrane potential (ΔΨm), citrate synthase activity, mtDNA copy number	High-resolution respirometry, ATP assays, enzyme activity assays, Qpcr	Enhanced cellular bioenergetics, improved muscle metabolism, superior meat quality, enhanced stress resilience, improved production efficiency	Abasht et al., 2016; Chen et al., 2022; Greene et al., 2025; Ouyang et al., 2022
Redox Resilience	SOD, GPx, catalase (CAT), total antioxidant capacity (T-AOC), malondialdehyde (MDA	Antioxidant enzyme assays, oxidative-stress profiling, lipid-peroxidation assays	Reduced oxidative injury, improved muscle integrity, enhanced meat color stability, improved shelf life, improved egg quality	Biabani et al., 2024; Calik et al., 2022; Chen et al., 2022; Deng et al., 2022; Elgendey et al., 2022; Mancinelli et al., 2023
Microbiome Stability	Alpha-diversity indices, *Lactobacillus* abundance, SCFAs, microbial functional pathways	16S rRNA sequencing, shotgun metagenomics, metabolomics	Improved nutrient utilization, enhanced gut integrity, balanced immune regulation, improved production performance and product quality	Bernard et al., 2024; Brugaletta et al., 2023a,b; Dai et al., 2022; He et al., 2023; Lan et al., 2025; Oakley et al., 2014
Metabolic Adaptability	Amino acid profiles, glucose, triglycerides, TCA-cycle intermediates, metabolomic signatures	LC–MS/MS, GC–MS, targeted and untargeted metabolomics	Efficient nutrient partitioning, improved feed efficiency, optimized growth, enhanced meat and egg quality	Beauclercq et al., 2018; Cui et al., 2024; Dermane et al., 2024; Ge et al., 2022; Greene et al., 2020
Immune Competence	IL-6, IL-10, TNF-α, IgA, IgG, heterophil:lymphocyte ratio	ELISA, hematology, flow cytometry, cytokine profiling	Enhanced disease resistance, balanced inflammatory responses, improved physiological robustness and production stability	Calik et al., 2022; Elbaz et al., 2023; Elokil et al., 2024; Emami et al., 2021; Gautam et al., 2024
Endocrine Regulation	Corticosterone, IGF-1, triiodothyronine (T3), thyroxine (T4)	ELISA, LC–MS/MS, endocrine profiling	Optimized growth, metabolic homeostasis, reproductive performance, and adaptive physiological regulation	Ahmed-Farid et al., 2021; An et al., 2025; Kim et al., 2021; Mei et al., 2025
Environmental Adaptation Capacity	HSP70, HSP90, body temperature, respiration rate, behavioral indices	qPCR, physiological monitoring, precision sensors, automated environmental monitoring	Improved thermal tolerance, enhanced welfare, increased productivity, and improved biological robustness	Bai et al., 2023;; Balthazar et al., 2025; Doornweerd et al., 2024; Elwakeel, 2025; Kim et al., 2024
Muscle Integrity Network	Creatine kinase, calpain activity, collagen characteristics, muscle-fiber morphology	Histology, proteomics, enzyme assays, microscopy	Improved tenderness, water-holding capacity, structural integrity, reduced incidence of wooden breast and spaghetti meat	Abasht et al., 2016; Beauclercq et al., 2017; Baldi et al., 2021; Greene et al., 2025; Gu et al., 2024
Egg Formation System	Ovocleidin-116, ovocalyxins, carbonic anhydrase, calcium transport proteins	Proteomics, transcriptomics, eggshell ultrastructure analyses	Improved shell strength, shell ultrastructure, albumen quality, yolk quality, and functional egg characteristics	Chang et al., 2024a,b; Feng et al., 2020, 2023; Cui et al., 2021, 2025; Fu et al., 2024
Integrated Adaptive Systems Trait Prediction of Poultry Quality (ASTPQ Index)	Integrated mitochondrial, redox, microbiome, metabolic, immune, endocrine, environmental, muscle, and egg-quality biomarkers	Multi-omics integration, AI, ML, systems biology, predictive analytics	Accurate prediction of poultry quality, biological resilience, adaptability, production efficiency, precision nutrition responses, and long-term robustness across diverse production environments	Cui et al., 2024; Ding et al., 2025; Gao et al., 2025a; Lan et al., 2025; Li et al., 2024

Footnote: The ASTPQ framework is proposed as an integrative systems-biology model linking molecular, physiological, microbial, environmental, and product-quality indicators for predictive poultry biology. ASTPQ, Adaptive Systems Trait Prediction of Poultry Quality; ATP, adenosine triphosphate; ΔΨm, mitochondrial membrane potential; mtDNA, mitochondrial DNA; GPx, glutathione peroxidase; CAT, catalase; T-AOC, total antioxidant capacity; MDA, malondialdehyde; IL, interleukin; TNF-α, tumor necrosis factor-α; IgA, immunoglobulin A; IgG, immunoglobulin G; IGF-1, insulin-like growth factor-1; T3, triiodothyronine; T4, thyroxine; HSP70, heat shock protein 70; HSP90, heat shock protein 90; qPCR, quantitative polymerase chain reaction; LC–MS/MS, liquid chromatography–tandem mass spectrometry; GC–MS, gas chromatography–mass spectrometry; ELISA, enzyme-linked immunosorbent assay; 16S rRNA, 16S ribosomal RNA.

## Future direction

Future advances in predictive poultry biology will depend on shifting from descriptive multi-omics integration toward mechanistic and predictive frameworks capable of explaining, forecasting, and guiding biological outcomes across diverse production environments. A central priority is the development of causal systems models integrating molecular, physiological, microbial, and environmental processes across temporal and biological scales, enabling reliable prediction of complex phenotypes and adaptive responses. Several fundamental scientific questions remain unresolved, including the relative value of high-dimensional multi-omics datasets versus strategically defined biomarker panels and the extent to which advanced AI systems can improve predictive performance while maintaining biological interpretability. Addressing these challenges will require comparative benchmarking across populations, environments, and production systems, alongside stronger integration of experimental validation and computational modeling. Emerging opportunities include longitudinal systems analyses, digital-twin frameworks, real-time biological monitoring, and adaptive predictive platforms capable of continuously integrating biological and environmental information. As these approaches mature, they are expected to enable a transition from retrospective characterization to anticipatory management strategies that enhance resilience, sustainability, animal welfare, and product quality in poultry production systems. The next phase of poultry systems biology will be defined by transforming biological complexity into mechanistic understanding, reliable prediction, and actionable intervention.

## Conclusion

Poultry systems biology has advanced understanding of meat and egg quality by demonstrating that performance, resilience, and product characteristics emerge from interconnected biological networks involving host genetics, metabolism, mitochondrial bioenergetics, oxidative regulation, endocrine signaling, immune function, microbiome ecology, and environmental influences. Multi-omics technologies have provided important insights into the mechanisms governing quality variation across production systems through the integration of genomic, transcriptomic, proteomic, metabolomic, microbiome, and phenotypic information. Contemporary research recognizes that meat and egg quality are systems-level phenotypes shaped by dynamic interactions among regulatory processes rather than by isolated molecular determinants. Integrated analytical frameworks facilitate the identification of biologically meaningful signatures associated with growth, reproduction, resilience, product quality, and environmental adaptation, thereby enabling predictive and mechanism-informed management. Current evidence supports a transition from descriptive to predictive biology, in which knowledge is integrated with advanced analytics, precision management, and decision-support technologies. Collectively, these advances establish the foundation for next-generation precision production systems that support evidence-informed breeding, nutrition, environmental management, health management, animal welfare, and quality management. The convergence of systems biology, precision agriculture, AI, and real-time production analytics is transforming poultry production into a predictive, adaptive, and biologically informed system. As scientific understanding continues to advance, integrating mechanistic biology with management innovation offers opportunities to enhance productivity, product quality, sustainability, animal welfare, and long-term resilience across global poultry production systems.

## CRediT authorship contribution statement

**Getahun Belay Mekonnen:** Writing – review & editing, Writing – original draft, Validation, Conceptualization.

## Disclosures

The authors declare that they have no known competing financial interests or personal relationships that could have appeared to influence the work reported in this paper.
